# Aryl Hydrocarbon Receptor-Dependent inductions of omega-3 and omega-6 polyunsaturated fatty acid metabolism act inversely on tumor progression

**DOI:** 10.1038/s41598-020-64146-6

**Published:** 2020-05-12

**Authors:** Sara Huerta-Yepez, Ana Tirado-Rodriguez, Mayra R. Montecillo-Aguado, Jun Yang, Bruce D. Hammock, Oliver Hankinson

**Affiliations:** 1 Department of Pathology & Laboratory Medicine, University of California, Los Angeles, CA 90095 USA; 2 Research Unit of Oncology Diseases. Hospital Infantil de Mexico, Federico Gomez, Mexico City, Mexico; 3 Department of Entomology and Comprehensive Cancer Center, University of California, Davis, CA 95616 USA; 4 Molecular Toxicology Interdepartmental Program and Department of Environmental Health Sciences, University of California, Los Angeles, CA 90095 USA

**Keywords:** Cancer microenvironment, Cancer prevention, Cancer, Diseases, Oncology

## Abstract

The Western diet contains a high ratio of omega-6 (ω6) to omega-3 (ω3) polyunsaturated fatty acids (PUFA). The prototypical aryl hydrocarbon receptor (AHR) ligand, 2,3,7,8-Tetrachlorodibenzo-p-dioxin (TCDD), induces CYP1 family enzymes, which can metabolize PUFA to epoxides. Mice fed ω3-rich or ω6-rich diets were treated with TCDD and injected subcutaneously with AHR-competent Hepa1-GFP hepatoma cells or AHR-deficient LLC lung cancer cells. TCDD reduced the growth rates of the resulting tumors in ω3-fed mice and inhibited their metastasis to the liver and/or lung, but had the opposite effects in mice fed ω6 PUFA. These responses were likely attributable to the corresponding PUFA epoxides generated in tumor cells and/or host, since many depended upon co-administration of a soluble epoxide hydrolase (EPHX2) inhibitor in males, and/or were associated with increases in epoxide levels in tumors and sites of metastasis. Equivalent effects occurred in females in the absence of EPHX2 inhibition, probably because this sex expressed reduced levels of EPHX2. The responses elicited by TCDD were associated with effects on tumor vascularity, tumor cell proliferation and/or apoptosis. Thus environmental AHR agonists, and potentially also endogenous, nutritional, and microbiome-derived agonists, may reduce or enhance cancer progression depending on the composition of dietary PUFA, particularly in females.

## Introduction

The aryl hydrocarbon receptor (AHR) binds a number of environmental pollutants, including 2,3,7,8-Tetrachlorodibenzo-ρ-dioxin (TCDD), polychlorinated biphenyls (PCBs), and certain polycyclic aromatic hydrocarbons (PAHs), some endogenous metabolites, including the tryptophan catabolite kynurenine, certain products generated by commensal microbes and dietary components, including compounds found in cruciferous vegetables^1–4^. After binding TCDD and other ligands, the AHR turns on the transcription of a large number of genes. CYP1A1, CYP1A2 and CYP1B1 are induced in many tissues in all mammals, and are also generally the most massively induced genes^5,6^. Interestingly, TCDD and other AHR agonists can both enhance or retard tumor growth in experimental mouse models, depending on context^7,8^.

**Figure 1:**
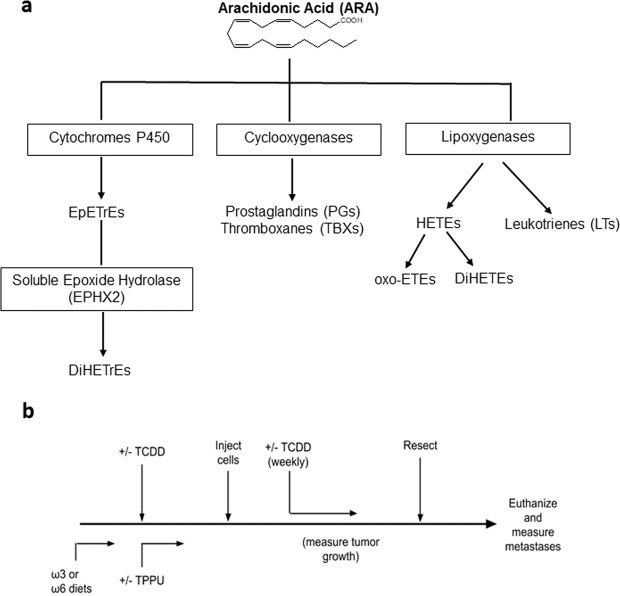
Simplified pathways of ARA metabolism (**a**), and generalized experimental protocol (**b**). EpETrEs (epoxyeicosatrienoic acids), DiHETrEs (dihydroxyeicosatrienoic acids), HETEs (hydroxyeicosatretraenoic acids), oxo-ETEs (oxo-eicosatetraenoic acids), DiHETEs (dihydroxytetraenoic acids).

High intake of omega-6 (ω6) polyunsaturated fatty acids (PUFA) such as arachidonic acid (ARA) and linoleic (LA) acid, characteristic of the western diet, is linked to a number of adverse health effects, including cancer. In contrast, the omega 3 (ω3) PUFAs, α-linolenic acid (ALA), eicosapentaenoic acid (EPA), and docosahexaenoic acid (DHA) are generally correlated with beneficial health effects^9^. PUFA are metabolized through three pathways: the “cyclooxygenase”, “lipoxygenase” and the lesser studied “cytochrome P450 epoxidation/hydroxylation” pathways (see Fig. 1a for the metabolism of ARA). PUFA epoxides are further metabolized to diols, which are generally considered less biologically active, by soluble epoxide hydrolase (EPHX2)^10^. Increasing the levels of epoxides of ω6 PUFA in mice stimulated tumor growth, angiogenesis and metastasis^11,12^. In contrast, systemic administration of epoxide derivatives of ω3 PUFA inhibited growth and metastasis of xenografted tumor cells^13^. Stimulation of arachidonic acid epoxidation within breast cancer cells increased their metastasis to the lung^14,15^. Synthesis of PUFA epoxides within the tumor cells and within the host can therefore independently impact tumor progression. No receptor(s) for PUFA epoxides has been unambiguously identified, however, and the mechanisms of their action are not clear.

CYP1 family members can metabolize PUFA *in vitro*^16–18^. To study whether such metabolism occurs *in vivo*, we measured 67 oxylipins (i.e. oxygenated metabolites of PUFA) in several organs of C57BL/6 wild-type or AHR knockout mice fed a chow diet three days after intraperitoneal injection of TCDD. TCDD increased the levels of a number of epoxides and diols of ARA, ALA, EPA and DHA in the liver and lung. TCDD also increased the levels of some of the hydroxylated metabolites of these PUFA. Where measured, TCDD increased the levels of the esterified metabolites in parallel with the corresponding free metabolites. TCDD did not increase oxylipin levels in AHR knockout mice, demonstrating that AHR mediates all or most of the effects of TCDD on the oxylipins^19,20^.

Since TCDD enhanced the cytochrome P450-dependent metabolism of ω3 and ω6 PUFA *in vivo*, we decided in the current study to investigate the potential effect of this metabolism on tumor progression.

## Results

### General experimental approach

The general outline of the experiments is shown in Fig. 1b. Particulars of the experimental methods are provided in the Methods section, while details of the individual experiments are described in the relevant paragraphs of the Results section. Mice were treated with or without TCDD and fed with experimental diets from three weeks prior to subcutaneous injection of tumor cells and throughout the remainder of the experiment. Tumor growth rate was measured, and when the tumors reached the same size in all experimental groups in a particular experiment, the tumors were resected. The resulting liver metastases were enumerated and extent of lung metastasis determined. (The number of surface metastases and the weight of the lung provided two measurements of lung metastasis.) The development of metastasis in this protocol is believed to correspond to reversal of tumor dormancy, and to represent a spontaneous model of metastasis^12^. A number of variables were addressed in our experiments:

- Cell Lines.
  Either the Hepa1-GFP cell line or the Lewis lung carcinoma (LLC) cell line was used.
- Mouse Strains.
  C57BL/6 strains were used because the C57BL/6 mouse is “AHR-responsive” (homozygous for the *Ahr*^*b-1*^ allele) and highly inducible by TCDD for the CYP1 family enzymes. Hepa1-GFP cells did not generate tumors after subcutaneous injection into wild-type C57BL/6 mice. We therefore utilized C57BL/6 *nu/nu* for the experiments with the Hepa1-GFP cells. LLC cells are syngeneic with the C57BL/6 mouse and we used the wild-type strain for these cells.
- Diets.
  Two different high ω6 diets, an isocalorific high ω3 diet (Supplementary Table S1), or normal mouse chow were used. ω6 rich diet 28 contained 1.3% PUFA had a theoretical ω6/ω3 ratio of 60:1, and an experimentally determined ω6/ω3 ratio of 38. These ratios resembled that in an extreme Western diet (Table 1). In certain experiments, we used ω6 rich diet 21, possessing a theoretical ω6/ω3 ratio of 20:1 and an experimentally determined ω6/ω3 ratio of 23 (Table 1). This diet more closely resembles the ω6/ω3 ratio in the typical western diet than does diet 28 (Stoll et al., 2001). Our ω3-rich diet (diet 29) contained 1% PUFA and had a ω6/ω3 ratio of 1.1:1 (theoretical) or 1.5:1 (experimental). This ω6/ω3 ratio is equivalent to the ratio recommended for the human by a panel of nutritionists^21^. The chow diet has a ω6/ω3 ratio of 5.8 and therefore is intermediate in this regard between our ω6 and ω3 diets. However most of the ω3 PUFA in the chow diet is in the form of alpha-linolenic acid rather than EPA and DHA, which comprise the bulk of the ω3 PUFA in our ω3-rich diet, 29 (Table 1). Importantly mice grew (or maintained their weights) equally on all the diets.

**Table 1:** Fatty Acid composition of diets.

	Diet 28 (high ω6)		Diet 29 (high ω3)		Diet 21 (high ω6)		Normal chow
	Theoretical	Experimental	Theoretical	Experimental	Theoretical	Experimental	Experimental
**Fatty Acids Information (g/kg diet)**							
Saturated Fatty Acids	48.2	44.2	50.2	43.9	48.4	43.8	14.3
Monounsaturated Fatty Acids	8.9	9.2	8.2	7.5	8.9	9.1	14.4
Polyunsaturated Fatty Acids	12.8	12.3	10.5	9.85	12.6	11.7	34.3
**Selected n6 FA**							
C18:2n6 Linoleic acid	12.6	3.5	4.7	5.75	11.9	12.14	29.7
C20:4n6 Arachidonic acid	0	0	0.3	0.15	0.3	0.15	0.15
Sum of n6 FA	12.6	13.3	5.0	5.9	12	12.4	30.0
**Selected n3 FA**							
C18:3n3 alpha-Linolenic acid	0.2	0.3	0.3	0.35	0.2	0.3	3.37
C20:5n3 Eicosapentaenoic acid (EPA)	0	0.1	2.3	1.75	0.2	0.1	1.02
C22:5n3 Docosapentaenoic acid	0	0	0.6	0.3	0.05	0	0.15
C22:6n3 Docosahexaenoic acid (DHA)	0	0	1.5	2.3	0.1	0.1	0.63
Sum of n3 FA	0.2	0.35	4.7	3.85	0.6	0.5	5.17
n6:n3 ratio	60.5	38.3	1.1	1.55	20.2	22.9	5.8
**Dietary Fat Sources, g/kg of diet**							
Coconut Oil	49.2		49.2		49.2		—
Fish Oil	0		14.3		1.2		—
Corn Oil	20.8		6.5		19.6		—
  We found that the levels of ω6 and ω3 PUFA in plasma rapidly changed within three days of the commencement of the diets, and remained relatively stable for a further 21 days (See Fig. 2a for the ω6/ω3 ratio at different time points and Supplementary Fig. S2 for the levels of individual PUFA). Furthermore, analysis of fatty acids twenty-four days after the initiation of feeding at the site of primary tumor formation (skin) and at the potential sites of metastases (lung and liver), showed that the levels of the PUFA reflected those in the respective diets (See Fig. 2b for the ω6/ω3 ratio in the different organs and Supplementary Table S2 for the levels of individual PUFA). The resulting ω6/ω3 ratios in the organs/tissues of the mice fed ω6-rich diet 21 were only slightly less than those in mice fed ω6-rich diet 28 except for skin, where the ω6/ω3 ratio was even greater in mice fed diet 21 (Fig. 2b and Supplementary Table S2). The ω6/ω3 ratios in the tissues/organs of mice fed the chow diet more closely resembled the ratio in the mice fed the ω6-rich diets than the ratio in mice fed the with the ω3-rich diet

**Figure 2:**
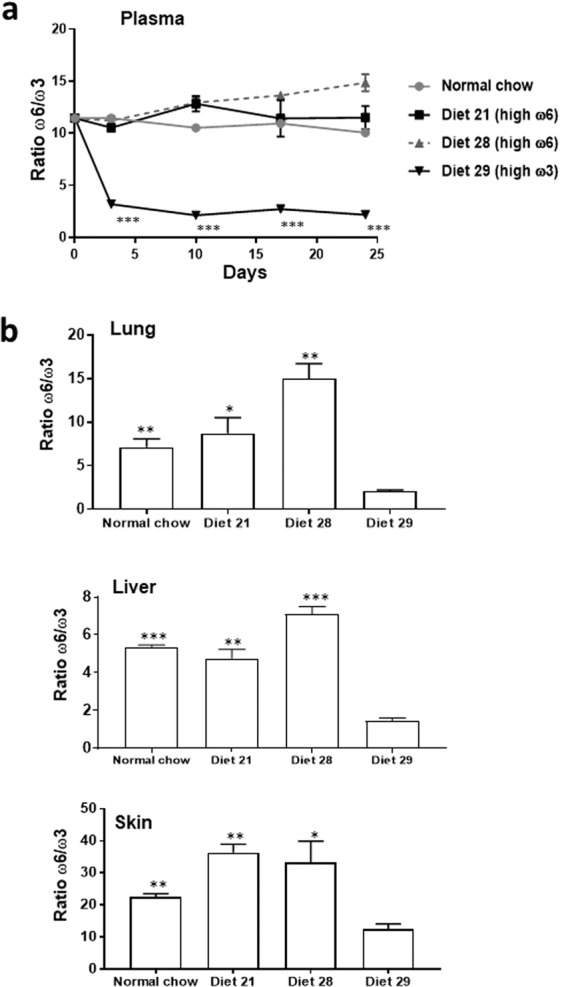
Ratio of ω6 to ω3 PUFA in mouse plasma/organs. (**a**) Ratio in mouse plasma after initiation of feeding of the diets, and (**b**) in mouse organs 24 days after initiation of feeding of the diets. *p < 0.05, **p < 0.01, ***p < 0.001 for diet 29 com*p*ared with the other diets. The data was analyzed with a two-tailed t test.
- Concentration of TCDD.
  The concentration of TCDD differed between the Hepa1-GFP and LLC experiments, as described in the relevant sections.
- Sex.
  Males were used in some experiments, and females in others.
- EPHX2 (soluble epoxide hydrolase) inhibitor.
  The presence or absence of the EPHX2 inhibitor, TPPU, represented a variable in some experiments.
- Oxylipin measurements.

In certain experiments we measured the levels of 67 to 75 oxylipins in the tumors or organs of the mice.

### Effect of TCDD on the growth of primary tumors arising from xenografted Hepa1-GFP cells and their metastasis to the lung in male immunocompromised mice fed either a ω3-rich or ω6-rich diet

We injected male nude C57BL/6 mice intraperitoneally with 4 µg/kg TCDD prior to injecting 10^6^ Hepa1-GFP cells subcutaneously, and injected the mice with 1 µg/kg TCDD weekly thereafter. 4 µg/kg TCDD maximally induces CYP1A1 and nearly maximally induces CYP1B1 in the livers of C57BL/6 mice^22^. Hepa1-GFP cells express the AHR and are highly inducible by TCDD for CYP1A1, moderately inducible for CYP1A2, and are not inducible for CYP1B1 (Supplementary Fig. S1). Hepa1-GFP expressed negligible levels of EPHX2 mRNA, less than 0.01% of the levels present in mouse liver, and fewer than 1 molecule of EPHX2 mRNA per 200 cells. This cell line allowed us to assess the effect of AHR expression in both the mouse host and the tumor cells to the effects of TCDD. In all these experiments we also treated the mice with the EPHX2 inhibitor, TPPU in their water. Since Hepa1-GFP cells do not express EPHX2, the effects of TPPU must be restricted to the mouse host. The following observations were made:

1. TCDD reduced the growth rate of tumors derived from Hepa1-GFP cells when the mice were fed the high ω3 diet (Fig. 3a).

**Figure 3:**
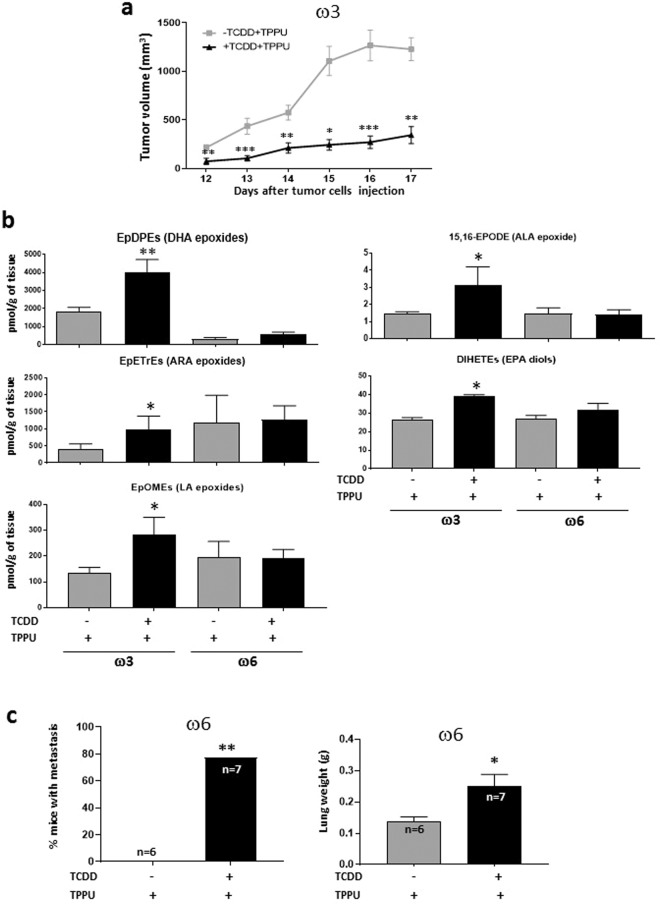
Effect of TCDD on Hepa1-GFP-derived tumors. (**a**) Effect of TCDD on tumor growth in mice fed a diet rich in ω3 PUFAs. Male C57BL/6 nude mice were fed with ω3-rich diet 29 and treated with or without TCDD (n = 10 per group). Both groups received TPPU. The mice were injected subcutaneously with Hepa1-GFP cells and tumor volume subsequently assessed. A representative experiment is shown of four independent experiments. Data were analyzed by the 2-tailed Mann-Whitney U test for comparison of two independent groups (**b**) Levels of oxylipins affected by TCDD treatment in tumors from mice fed the ω3-rich or ω6-rich diet plus TPPU. Up to five tumors were analyzed per experimental group. Data were analyzed by a two-tailed t test. **(c**) Effect of TCDD on lung metastasis in mice fed an ω6-rich diet. Male C57BL/6 nude mice were fed diet 28, treated or untreated with TCDD, and treated with TPPU. Tumors were resected four to six weeks after the injection of Hepa1-GFP cells. After a further five to six weeks the mice were euthanized and the frequency of mice with lung metastases determined and lungs weighed (analyzed by Fisher’s exact test and a two-tailed t test, respectively). The data represents the summation of two independent experiments. 15 mice were resected per experimental group. The numbers of mice surviving to the point when metastases could be measured are shown. Levels of significance are indicated as follows: *p < 0.05, **p<0.01, ***p<0.001.
2. TCDD did not consistently affect the rate of tumor growth in mice fed the high ω6 diet (diet 28). Data not shown.
3. 67 oxylipins were measured in the tumors tissue (Fig. 3b). The only metabolites that were found to be significantly affected in tumors from TCDD-treated ω3-fed mice were the epoxides of DHA (ω3), ARA (ω6), LA (ω6) and ALA (ω3) and the diols of EPA, which were all elevated in the tumors of ω3-fed mice. The increase in the levels of the DHA epoxides (EpDPEs) were considerably greater than those for the other epoxides. These results are consistent with the notion that the inhibitory effect of TCDD on the growth of tumors in ω3-fed mice is due to the generation of the corresponding epoxides. Note that the epoxides in the tumors could have been generated by both the tumor calls and tumor-associated host cells.
4. The only oxylipin increased by TCDD in ω6-fed mice was the lipoxygenase product of linoleic acid (ω6), 9-oxo-ODE). This data is congruent with our observation that TCDD did not enhance tumor growth in mice fed the ω6-rich diet.
5. The levels of all 67 oxylipins in the four treatment groups are presented in Supplementary Table S3. The levels of the ω3 metabolites in the tumors were generally considerably greater in those from ω3-fed mice than ω6-fed mice, and the levels of the ω6 metabolites were generally greater in the tumors from the ω6-fed mice, reflecting the concentrations of the PUFA in the diets.
6. Tumors had been resected at various times after primary tumor initiation (between 26 and 33 days) to ensure that average size of the tumors did not vary appreciably among the various experimental groups. (See Supplementary Table S4 for the size of the tumors in ω6-fed mice at the time of resection). On average 43% of the resected mice could be analyzed for metastases after resection: The remainder died prematurely, were euthanized for health reasons, or were euthanized because of reappearance of the primary tumor. No lung metastases were observed in mice fed with either the ω3 or ω6-rich diets in the absence of TCDD. However, TCDD treatment significantly increased the number of mice with lung metastases and also lung weight in mice fed the ω6-rich diet (Fig. 3c).
7. Since no lung metastases were obtained in mice fed the ω3-rich diet in the absence of TCDD we were not able to assess whether TCDD reduced lung metastasis in these mice.
8. No liver metastases were found in any mice.

These experiments therefore demonstrated that when TCDD activates the AHR in both the host and tumor it (i) increases the levels of ω3 epoxides in the tumors of ω3-fed mice and inhibits their growth, and (ii) enhances lung metastasis in ω6-fed mice (by an unknown mechanism). The results of our tumorigenesis and metastasis experiments are summarized in Table 2.

**Table 2:** Summary of the effects of TCDD.

Cell line	Mouse host	Diet	TPPU	Effect of TCDD on:		
				Tumor Growth	Metastasis:	
					Lung	Liver
Hepa1-GFP (AHR+)	Male C57BL/6 nu/nu	ω3	+	↓		
		ω6	+		↑	
LLC (AHR-)	Male C57BL/6	ω3	+	↓	↓	↓
			−	−	−	
		ω6	+	↑	↑	↑
			−	−	↑	↑
	Female C57BL/6	ω3	+	↓	↓	
			−	↓	↓	
		ω6	+	↑	↑	
			−	↑	↑	

### Effect of TCDD and the ω3- and ω6- rich diets on the growth and metastasis of xenografted LLC cells in male mice

For these experiments, we injected C57BL/6 male mice with 40 μg/kg of TCDD intraperitoneally prior to subcutaneous injection of 2 × 10^5^ LLC cells. This TCDD dose is frequently used in studies with mice, is not overtly toxic, and leads to sustained levels of CYP1A1 for at least a week^23^. Importantly, we previously showed that CYP1A1 and CYP1B1 mRNA levels were greatly increased in the liver and lung by this dose of TCDD, and CYP1A2 mRNA was increased in the liver. Furthermore, our studies on oxylipin levels were obtained with this dose^19,20^. The mice were also treated with 10 µg/kg TCDD at weekly intervals after the initial dose. LLC cells expressed less than 0.01% of the EPHX2 mRNA levels present in mouse liver, and fewer than 3 molecules of EPHX2 mRNA per 200 cells. These experiments provided us with the opportunity to study the effects of TCDD on the host in the absence of any direct effects on the tumor cells. The following data were obtained:

1. TCDD reduced the growth rate of LLC-derived tumors in male mice fed the high ω3 diet in the presence of the epoxide hydrolase inhibitor, TPPU. The growth of tumors was not inhibited in the absence of TPPU (Fig. 4a).

**Figure 4:**
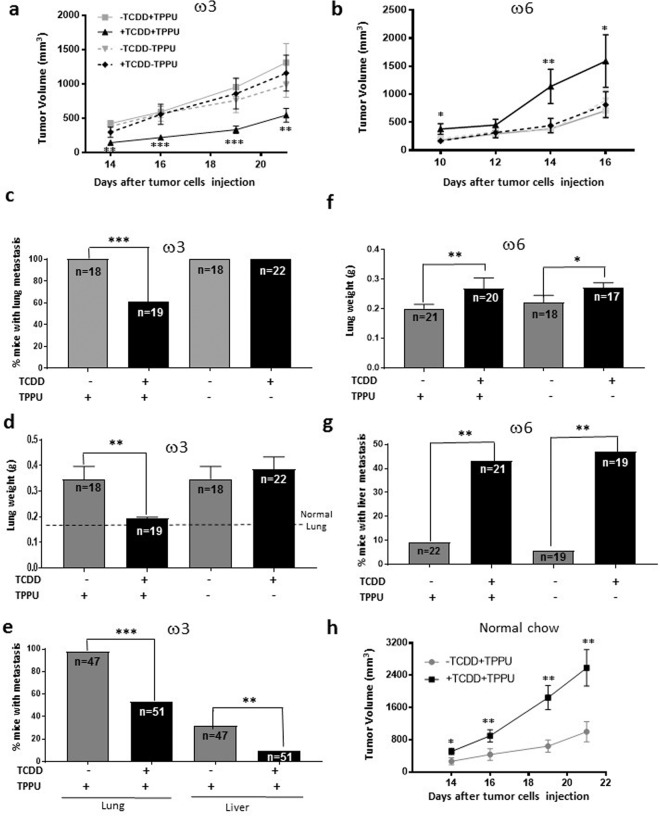
Effect of TCDD on the growth and metastasis of LLC-derived tumors in male mice fed the ω3-rich or ω6 diet or chow in the presence or absence of the soluble epoxide hydrolase inhibitor, TPPU. (**a**) A representative tumor growth experiment with 8 to 10 mice per group is shown of four (+TPPU) or two (-TPPU) independent experiments in ω3-fed mice. (**b**) A representative experiment is shown of two independent experiments in ω6-fed mice (10 mice per experimental group). (**c,d**) Frequencies of mice with lung metastases and lung weights in male mice fed the ω3-rich diet 29 in the presence and absence of TPPU. The data represent the summation of three independent experiments. 32 mice were resected per experimental group. The numbers of mice surviving to the point when metastases could be measured are shown. (**e**) Lung and liver metastasis of LLC-derived tumors. The data represent the summation of results from six experiments that are different from those represented in Fig. c,d 76 mice were resected per experimental group. (**f,g**) Lung weights and frequencies of mice with liver metastases in mice fed the ω6-rich diet. The data represent the summation of two independent experiments. 24 to 27 mice were resected per experimental group. (**h**) A representative tumor growth experiment with mice fed the chow diet, and randomized into four groups (n = 10/group): treated or untreated with TCDD and provided or not provided with TPPU, as indicated. A representative experiment is shown of two independent experiments.
2. Opposite to its effect in mice fed the high ω3 diet, TCDD accelerated the growth of LLC-derived tumors in male mice fed the high ω6 diet 21 when they were treated with TPPU (Fig. 4b).
3. The above data suggest that the effects of TCDD on tumor growth are mediated at least in part, by epoxides of the PUFA.
4. In the absence of treatment with TCDD, the growth rate of LLC-derived tumors in male mice fed the high ω3 diet did not differ from the growth rate of mice fed the high ω6 diet (Supplementary Fig. S3). Thus our results were not confounded by any differential growth rates of tumors in mice fed the high ω3 or ω6 diets in the absence of TCDD.
5. LLC-derived tumors in male mice fed the ω3-rich diet were resected when they were approximately 3,000 mm^3^ in size (Supplementary Table S4). In individual experiments, tumors were excised various times after tumor cell injection (varying between 19 and 34 days) to ensure that the average size of the tumors did not vary appreciably between different experimental groups. Unlike the case with mice injected with Hepa1-GFP cells, the great majority of male mice fed the ω3-rich diet and treated with (or without) TPPU in the absence of TCDD were found to have lung metastases. This number was significantly decreased in mice treated with TCDD and TPPU (Fig. 4c). Lung weight was also decreased by TCDD in these mice (Fig. 4d). TCDD did not inhibit the development of metastases in the absence of TPPU. These data suggest that the TCDD-mediated reduction in lung metastasis occurring in ω3-fed mice is dependent upon the generation of the corresponding epoxides.
6. Figure 4e presents a summation of the results from a total of six separate experiments (that are different from those represented in Fig. 4c,d) in which we tested the effect of TCDD on metastasis in ω3-fed mice in the presence of TPPU. With this increased number of mice, we were able to observe a significant decrease in the number of liver as well as lung metastases in mice treated with TCDD.
7. Since the great majority of mice developed lung metastases even in mice untreated with TCDD, we could not assess whether TCDD increased the number of lung metastases in ω6-fed mice. However, we did observe increased lung weight in TCDD-treated ω6-fed mice (reflecting an increase in metastatic load), and interestingly this occurred in both TPPU-treated and untreated animals (Fig. 4f).
8. TCDD increased the number of liver metastases in ω6-fed mice, and this also occurred both in mice treated or untreated with TPPU (Fig. 4g). (In order to minimize lethality, we resected the LLC-derived tumors in the ω6-fed mice when they were smaller {2,500 mm^3^; Supplementary Table S4} than the corresponding tumors in ω3-fed mice, and also euthanized the former mice sooner after resection than the latter mice. This probably explains why the lung weights and frequency of mice with liver metastases were lower in the ω6-fed mice untreated with TCDD compared with these parameters in the equivalent ω3-fed mice.
9. The above data suggest either that sufficient levels of ω6 epoxides were generated for metastasis to occur in the lungs and livers of ω6-fed mice even in the presence of active EPHX2, or that other metabolites of ω6 PUFA are responsible for enhancing metastasis.
10. The levels of 77 and 75 oxylipins were measured in the lungs and livers, respectively, of male C57BL/6 mice fed with either the ω3-rich diet (diet 29) or the ω6-rich diet (diet 21) for 4 weeks, maintained with TPPU, and injected intraperitoneally with 40 µg/kg TCDD or vehicle (1,4-dioxane) four days prior to euthanasia.
11. A large number of oxylipins were increased by TCDD treatment in the lungs of mice fed the ω6 diet (Fig. 5). Of note, TCDD increased the levels of the epoxides (EpETrEs) and diols (DiHETrEs) of the ω6 PUFA, ARA, and to a much lesser degree, the epoxides (EpETEs) and diols (DiHETEs) of the ω3 PUFA, EPA. These data are consistent with the notion that the enhancement of metastasis to the lungs of LLC cells in ω6-fed mice depended upon the generation of ARA epoxides in this organ. However, 11,12,15-TriHETrE, and many lipoxygenase metabolites of ARA: HETEs, oxoeicosatetraenoic acids {oxo-ETEs, 6-trans-leukotriene B4 (6-trans-LTB4), and lipoxygenase A4 were also increased by TCDD treatment in the lung, as were several cyclooxygenase products of ARA (thromboxane B_2_, and all five prostaglandins that were measured: PGD_2_, PGE_2_, PGF_2α_, PGJ_2_, and keto-PGF_1α_), entertaining the possibility that one or more of these metabolites may contribute to the stimulatory effect of TCDD on metastasis in the lung of ω6-fed mice.

**Figure 5:**
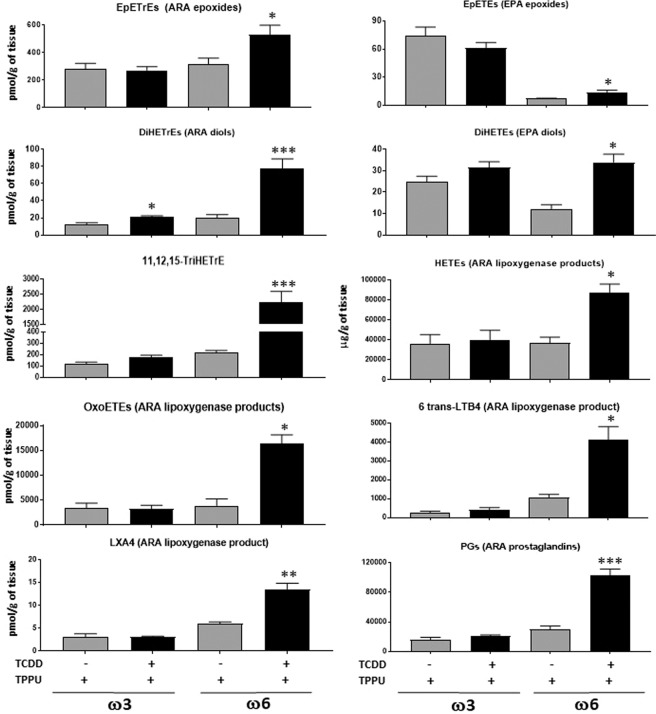
Oxylipins affected by six day treatment with 40 µg/kg TCDD and TPPU in the lung of mice fed with ω3-rich diet 29 or ω6-rich 21. Five mice were analyzed per experimental group. TriHETrE (Trihydroxyeicosatrienoic acid). Oxylipins levels are in pmol/gram of tissue.
12. We did not observe any pronounced changes in lung oxylipin levels induced by TCDD in ω3-fed mice that might suggest any roles in mediating the inhibition by TCDD of LLC metastasis in ω3-fed mice.
13. The levels of all 77 oxylipins in the lungs of the four experimental groups of mice are presented in Supplementary Table S3.
14. In the liver, TCDD increased the levels of EPA and DHA epoxides and DHA diols in the ω3-fed mice, consistent with the notion that ω3 epoxides are responsible for the decrease in liver metastases induced by TCDD in mice fed the ω3 diet (Fig. 6). TCDD treatment also lead to less marked increases in ARA and LA diols, 11,12,15-TriHETrE, and EPA-derived prostaglandins in the ω3-fed mice.

**Figure 6:**
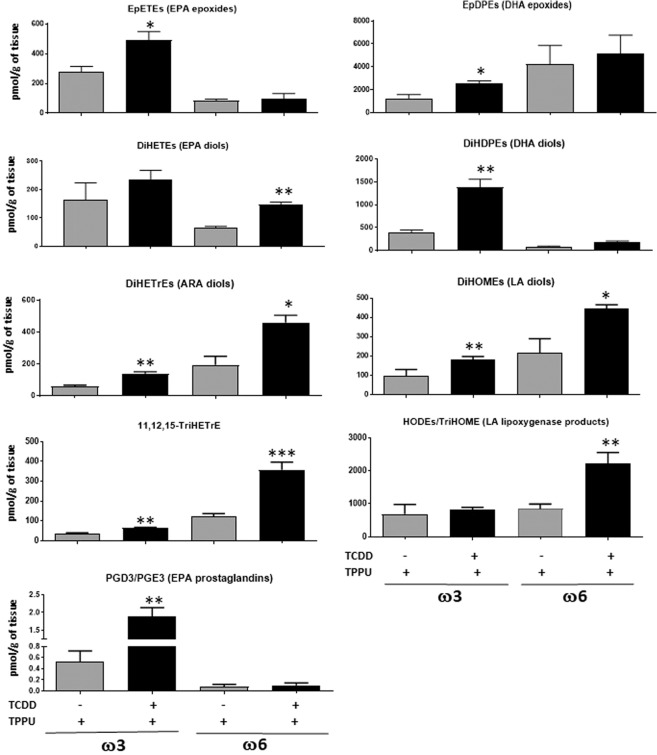
Oxylipins affected by six day treatment with 40 µg/kg TCDD and TPPU in the liver. Five mice were analyzed per experimental group. DiHETEs, DiHDPEs, DiHETrEs, DiHOMEs {dihydroxyoctadecaenoic acids} (Diols of EPA, DHA, ARA and LA respectively). HODEs {hydroxyoctadecaenoic acids} and TriHOMEs {trihydroxyoctadecaenoic acids} (Dihdroxy and trihydroxy metabolites of LA).
15. TCDD treatment in ω6-fed mice increased the liver levels of the diols of ARA, LA and EPA, 11,12,15-TriHETrE, and the hydroxy lipoxygenase metabolites of LA (hydroxyoctadecaenoic acids {HODEs} and trihydroxyoctadecaenoic acids {TriHOMEs}). It is possible that one or more of these cytochrome P450 metabolites, or their epoxide precursors, and/or lipoxygenase metabolites contributed towards the stimulatory effect of TCDD on the metastasis of LLC cells in the livers of ω6-fed mice.
16. The data for all the oxylipins in liver are presented in Supplementary Table S3.
17. The levels of the ω6 metabolites were in general considerably greater in the livers of the ω6-fed mice, and the ω3 metabolites were greater in the livers of the ω3-fed mice, as expected. (Supplemental Table S3).
18. We also investigated the effects of TCDD on the rate of tumor growth and on the level of metastasis after tumor resection in mice fed a normal chow diet, since most mouse experiments are performed with this diet. Similar to the results obtained with the ω6 diet, and congruent with our observations that the chow diet generated ω6/ω3 ratios in the plasma and organs similar to those of ω6-fed mice, TCDD accelerated the growth rate of male mice fed the chow diet in the presence of TPPU (Fig. 4h). Equivalent results were obtained in two experiments.

In summary, these data demonstrate that TCDD inhibits tumor growth and lung and liver metastasis of AHR-deficient LLC cells in mice fed an ω3-rich diet, but stimulates these parameters in ω6-fed mice, and provides evidence that these effects are mediated by the corresponding epoxides, although other metabolites of the PUFA may also be involved.

### Effect of TCDD and the ω3- and ω6-rich diets on the growth and metastasis of xenografted LLC cells in female mice

1. TCDD reduced the growth rate of LLC-derived tumors in female C57BL/6 mice fed with the ω3-rich diet, as with male mice. However, in contrast to male mice, this reduction also occurred in the absence of TPPU (Fig. 7a)

**Figure 7:**
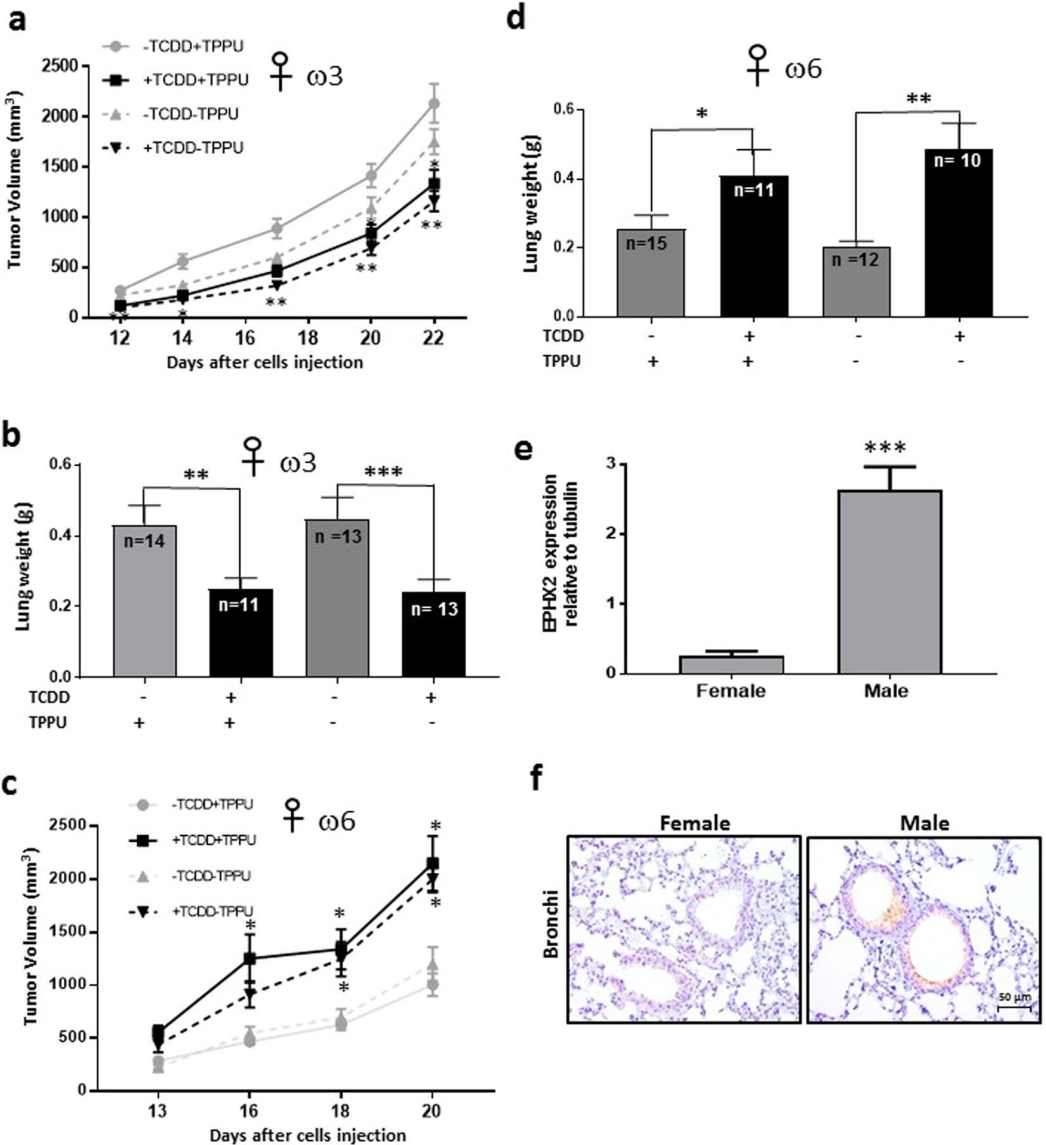
Effect of TCDD on the growth and metastasis of LLC-derived tumors in female mice. Female C57BL/6 mice were fed ω3-rich diet 29, provided with or without TPPU, and either treated or untreated with TCDD. (**a**) Tumor volume was assessed after injection with LLC cells (n = 14 to 21/group). (**b**) Lung weights were determined approximately five weeks after resection of tumors of approximately 3,700 mm^3^ in size (see Supplemental Table S4d). The data in a and b are each derived from two independent experiments. (**c**) Female mice were fed with ω6-rich diet 28, and tumor growth was determined (n = 9 to 15 per group). (**d**) Lung weight was measured after resection of tumors approximately 2,400 mm^3^ in size. (See Supplementary Table S4e.) The data in c and d are each derived from two independent experiments. *p < 0.05, **p < 0.01, *p < 0.001 compared with the corresponding –TCDD groups. (**e**) The levels of EPHX2 mRNA were evaluated in normal lung tissue from female and male mice (n = 4/group) fed omega 3-rich diet 29 by RT-PCR (mean ± SEM, ***p = 0.001). (**f**). EPHX2 protein was detected in the same mice by immunocytochemistry.
2. TCDD decreased the degree of lung metastasis after tumor resection (as determined by lung weight) in female mice fed the ω3-rich diet, and unlike in male mice, this also occurred in the absence of TPPU as well as in its presence (Fig. 7b).
3. TCDD increased the growth rate of LLC-derived tumors in female mice fed the ω6-rich diet and again, in contrast to males, these responses occurred in the absence of TPPU (Fig. 7c).
4. TCDD enhanced the degree of lung metastasis (as determined by lung weight) in ω6-fed female mice both in the presence and absence of TPPU (Fig. 7d), similarly to males. In order to minimize lethality, we resected the LLC-derived tumors in the ω6-fed mice when they were smaller {~2,400 mm^3^, Supplementary Table S4e} than the corresponding tumors in ω3-fed mice {3,700 mm^3^, Supplementary Table S4d}), and also euthanized the former mice sooner after resection than the latter mice. This probably explains why lung weights were lower in the ω6-fed mice untreated with TCDD compared with the equivalent ω3-fed mice.
5. EPHX2 mRNA levels were 28-fold lower in the lungs of female than male mice fed ω3-rich diet 29 (Fig. 7e), and females also expressed considerably lower levels of EPHX2 protein in the lung than males as determined by immunohistochemical analysis (Fig. 7f). This is consistent with a recent report that estrogen/estrogen receptor-dependent methylation of the *Ephx2* gene downregulates expression of this gene in mouse tissues and human cells^24^.

Thus all responses to TCDD in female mice occurred in the absence of TPPU. This is important because this represents a more real-world setting than the inclusion of an EPHX2 inhibitor. The reduced expression of EPHX2 in females provides a possible explanation for our observation that all responses to TCDD in females occurred in the absence of TPPU.

### Effect of TCDD on vascular cell density, VEGF and apoptosis in LLC-derived tumor tissue and tumor-associated tissues, and on plasma VEGF in male mice fed either an ω3-rich or an ω6-rich diet

In order to investigate the means whereby TCDD differentially affected tumor growth and metastases in male mice fed the high ω3 or the high ω6 diet, we assayed several parameters associated with these processes in C57BL/6 wild-type mice fed with either diet 29 or diet 31, treated with or without TCDD and with or without TPPU, and injected with LLC cells. The following results were obtained:

1. TCDD treatment did not increase the levels of the CYP1A1 protein in tumor tissues from mice fed either the ω3 or ω6 diet, consistent with the observation that cultured LLC cells are not inducible for CYP1A1 (Supplementary Fig. S4a,b).
2. TCDD did increase the levels of CYP1A1 in non-metastatic, but not non-metastacic segments of the lung from these same mice, confirming that our TCDD treatment protocol is effective at inducing this enzyme in mice (Supplementary Fig. S4c,d).
3. TCDD did not affect the levels of EPHX2 in the lungs of either ω3- or ω6-fed mice (Supplementary Fig. S5), consistent with our previous observations on EPHX2 mRNA expression in mice fed with chow^19^.
4. TCDD decreased both blood vessel density and VEGF levels in tumors from mice fed the ω3 diet but only when they were also treated with TPPU (Fig. 8a,b).

**Figure 8:**
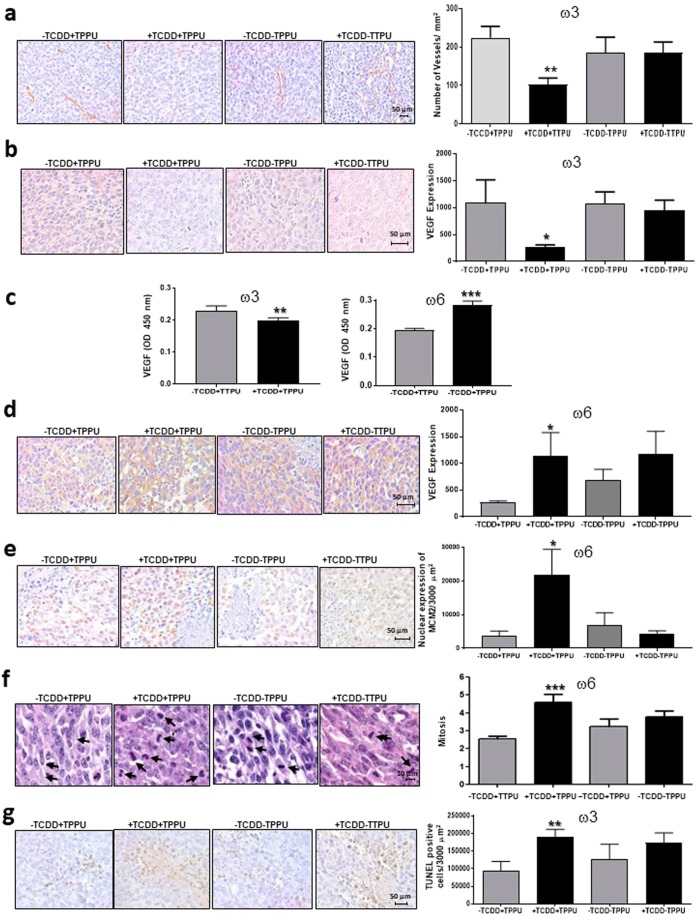
Blood vessels, VEGF levels, proliferation and apoptosis in LLC-derived tumors. Blood vessel density (**a**) and VEGF levels in LLC-derived tumors (**b,d**) and plasma (**c**). MCM2 levels (**e**) and mitotic indices (**f**) in the nuclei of LLC-derived tumors of ω6-fed male mice. Apoptosis in LLC-derived tumors of ω3-fed male mice (**g**). Data are representative of two or three independent experiments. *p < 0.05, **p < 0.01 compared with the corresponding –TCDD group.
5. In the presence of TPPU, TCDD decreased VEGF levels in the serum of mice fed the ω3-rich diet (Fig. 8c). Thus the effect of TCDD plus TPPU in mice fed the high ω3-diet may be partly mediated by a decrease in angiogenesis occurring as a result of locally decreased levels of VEGF.
6. TCDD increased VEGF levels in the serum of mice fed the ω6 diet (Fig. 8c), and increased VEGF expression in the tumors of ω6-fed mice (Fig. 8d), suggesting that increased VEGF may be at least partly responsible for the effect on TCDD in the ω6 diet.
7. TCDD increased the expression levels of the cell proliferation marker MCM2 and also the mitotic index in tumors derived from mice fed the ω6 rich diet (Fig. 8e,f) suggesting that an increase in tumor cell proliferation contributes to the stimulatory effect of TCDD in the presence of the ω6 diet.
8. TCDD increased apoptosis in the tumor tissue from mice fed the ω3-rich diet in the presence of TPPU (Fig. 8g), suggesting that apoptosis may contribute to TCDD’s effect on tumor growth under these conditions.

## Discussion

Using two tumor transplantation models, we demonstrate that TCDD generally retards primary tumor growth and metastasis when mice are fed an ω3-rich diet, but enhances these parameters in mice fed ω6-rich diets. This represents a novel process whereby TCDD (and presumably other AHR ligands) can impact tumor progression. Our studies also break new ground in showing effects of TCDD on both tumor growth and metastasis. Certain of the effects of TCDD were observed in nude mice, indicating that they do not depend upon a T cell-dependent immune response. It is likely that PUFA epoxides are primarily responsible for the effects of TCDD on tumor progression. As indicated by our following observations:

- In combination with an EPHX2 inhibitor, TCDD inhibited the growth of Hepa1-GFP derived tumors in ω3-fed male mice and also increased the levels of ω3 PUFA epoxides in these tumors.
- In male mice the negative effects of TCDD on the growth and lung metastasis of tumors derived from the AHR-negative LLC cells in mice fed the ω3 diet and the positive effect of TCDD on LLC tumor growth in ω6-fed mice occurred only when the mice were fed an EPHX2 inhibitor.
- TCDD decreased metastasis to the livers of LLC-derived tumors in ω3-fed male mice in the presence of TPPU, and increased the levels of ω3 epoxides and diols in the livers of equivalently treated mice.
- TCDD increased lung metastasis of LLC-derived tumors in ω6-fed and TPPU-treated male mice and also increased the levels of ARA epoxides in the lungs of such mice.
- TCDD treatment in ω6-fed mice increased liver metastasis and also the levels of the diols of ARA, LA and EPA in the liver.

Nevertheless, it should be noted that certain PUFA metabolites other than epoxides and diols were also increased in some of the above experiments, opening up the notion that they may also contribute to the observed effects of TCDD.

As with male mice, TCDD inhibited tumor growth and lung metastasis of LLC-derived tumors in ω3-fed female mice and enhanced tumor growth and lung metastasis in ω6-fed female mice. However, these responses occurred even in the absence of TPPU, in contrast to the situation in male mice (except for lung metastasis in ω6-fed male mice, which also occurred in the absence of TPPU). This suggests either that non-epoxide metabolites of the PUFA are responsible for effecting these changes in tumor progression, or more likely, that because of reduced levels of EPHX2 in females, TCDD treatment induces sufficient increases in PUFA epoxides even in the absence of EPHX2 inhibition to impact tumor growth and metastasis. Most importantly, the lack of a requirement for TPPU to see effects of TCDD in female mice reflects a real-world situation.

The positive effects of TCDD on the growth of LLC tumors in mice fed the chow diet resembled its effects in mice fed the ω6-rich diet, reflecting the similar levels of the ω3 and ω6 PUFA generated by these diets in the tissues of the host. Since most studies on the effects of AHR agonists in mice have been performed on animals fed a chow diet, it is likely that any resulting PUFA metabolism that occurred was stimulatory towards tumor progression.

Of interest, TCDD and other PCDDs can increase the levels of PUFA in the liver^25,26^ and such increases may further enhance the role of the metabolism of these PUFA in tumor growth and metastasis.

Our data indicate that that the effects of TCDD on metastasis in the presence of the ω3 and ω6 diets occur independently of its effects on tumor growth, consistent with the observations of other investigators with regard to the corresponding effects of the PUFA epoxides^11–13,15^ and suggesting that TCDD impacts the metastatic cascade directly under these circumstances.

We investigated various parameters associated with tumor growth and metastasis in tumors and/or stromal cells in the LLC experiments. Previous studies indicated that the VEGF pathway may contribute the effects of ω3 epoxides on tumor progression^13^. In concordance with these observations, we found that in mice fed the ω3-rich diet, TCDD decreased plasma VEGF levels, and both VEGF expression and blood vessel density in LLC-derived tumors, suggesting that the VEGF pathway may contribute to the effects of TCDD that we observed. We also observed that apoptosis was increased by TCDD (+TPPU) treatment in LLC-derived tumors in mice fed the high ω3 diet, suggesting that alterations in apoptotic rates may contribute to the effects we observed in the male mice fed this diet. TCDD increased plasma VEGF levels and VEGF levels in the LLC-derived tumors of mice fed the high ω6 diet, suggesting that increased VEGF levels may contribute to the stimulating effect of TCDD on tumor growth, and tumor cell proliferation and metastasis that we observed in mice fed the ω6 diet (plus TPPU).

Our observations are potentially relevant to the etiology of several types of human cancer. For example, environmental exposure to PAHs (e.g. via tobacco and open-fire smoke and smog), which are potent AHR agonists, and occupational exposure to these compounds (e.g. in coke oven workers) are known to cause lung, skin, gastrointestinal, and bladder cancer^27,28^. PAHs generated in barbecued meat can induce CYP1 family members in the small intestine and liver of humans^29^ that thereby metabolically activate the PAHs themselves and also carcinogenic heterocyclic amines in the cooked meat^30^. Natural products, including phytochemicals found in common vegetables, and also certain products generated by commensal microbes, are potent AHR agonists^1,2^. The endogenous tryptophan metabolite, kynurenine (or one or more of its metabolites^31^) can promote human glioblastoma via activation of the AHR^32^. Importantly, the AHR is also constitutively active and overexpressed in many types of cancer, and CYP1A1 and CYP1B1 are overexpressed in many cancers^4,33–35^. It is very possible, therefore, that a high ω6 consumption (as in a typical Western diet) will exacerbate progression of the tumors in the above situations, while a high ω3 diet may be protective.

EPHX2 is the principal enzyme involved in the further metabolism of PUFA epoxides. However, certain observations indicate that EPHX2 may have a limited effect on the levels of PUFA epoxides during the development of certain tumors, in certain individuals, or in particular contexts. Thus, EPHX2 expression diminishes in many types of tumors as they grow, is diminished in tumor-associated endothelial cells, is suppressed in metastases, and is eliminated or reduced in several types of human cancers and frequently also in the adjoining normal tissue^10,12,36^. Furthermore, polymorphisms in the *EPHX2* gene lead to great differences in soluble epoxide hydrolase activity in the human^37^. The ratio of PUFA diols to epoxides declines substantially in the plasma of rats shortly after a meal, suggesting a decline in EPHX2 activity^38^. Hypoxia diminishes EPHX2 expression in mouse lung^39^. Importantly, we observed that TCDD inhibited tumor growth and lung metastasis in female mice fed the ω3 diet and enhanced these parameters in female mice fed the ω6 diet even in the absence of TPPU. These effects may be related to the reduction in levels of EPHX2 in female mice that we observed, which are likely mediated by estrogen. The absence of an EPHX2 inhibitor reflects a real-world setting, and the above data suggests that premenopausal women may be particularly responsive to the effects (positive and negative) of dietary PUFA and AHR agonists on tumor growth and progression. Interestingly, women are more capable than men of metabolizing ALA to EPA and DHA^40^, and CYP1A1 and CYP1B1 expression in the lungs of women smokers (i.e. under inducing conditions) is considerably greater than in the lungs of smoking men^41,42^, further suggesting that the effects of dietary ω3 PUFA may be more effective in inhibiting AHR-dependent cancer progression in females than males.

In conclusion, we show that TCDD impacts the growth and metastasis of tumors in either a positive or negative way depending on the levels of ω3 and ω6 PUFA in the diet. Our studies therefore reveal a novel mechanism whereby AHR activation as well as CYP1 enzymes can impact the progression of cancer. The role of the CYP1 enzymes in the development and progression of cancer has hitherto focused on their ability to metabolize PAH and other environmental carcinogens. Our studies indicate that the ability of CYP1 enzymes to metabolize PUFA can also contribute to their cancer-related activities. Our studies furthermore point to benefits of a high ω3 PUFA diet. Certain cold water fish represent the main dietary source of DHA and EPA, and these represent a limited resource. However, it is encouraging that efforts are being pursued to bioengineer plants and microbes to produce DHA and/or EPA^43^.

## Methods

### Chemicals

1-Trifluoromethoxyphenyl-3-(1-propionylpiperidin-4-yl) urea (TPPU) was synthesized as described^44^. Stock solutions were prepared in Polyethylene Glycol 400 at 6.9 mg/L and diluted 1 in 1,000 in the water provided to the mice. TCDD dissolved in 1,4-dioxane was obtained from AccuStandard (New Haven, Connecticut). Mice were injected intraperitoneally with 10 μL of appropriate dilutions of this stock solution.

#### Cell lines and cell culture assays

The murine hepatoma cell line Hepa1-GFP was generated in our laboratory from Hepa-1c1c7 cells^45^ by infection with the pMSCV-ires/Green Fluorescent Protein (GFP) retroviral expression vector described previously^46^. The LLC cell line (ATCC #CRL-1642) was purchased from the American Type Culture Collection (Manassas, VA). Cell lines were cultured in alpha Minimal Essential Medium (α- MEM) (Gibco, Carlsbad, CA), supplemented with 10% fetal bovine serum (FBS, Omega Scientific, Tarzana, CA) and penicillin-streptomycin and Fungizone (Gibco). Cells for *in vivo* injections had greater than 90% viability and were harvested from sub-confluent cultures using 0.05% trypsin-EDTA, then neutralized with culture medium at 4 °C and washed 3 times in serum-free alpha medium before re-suspension in serum-free alpha medium.

### Mouse husbandry and feeding protocol

C57BL/6 wild-type or nude (B6.Cg-*Foxn1 nu/J*) mice five weeks old were purchased from the Jackson Laboratory (Bar Harbor, Maine). Mice were housed up to five per cage in independently ventilated cages at a constant temperature (20~22 °C) and a 12-h light/12-h dark cycle in a specific pathogen-free environment in the animal facility at UCLA. The mice were maintained on the NIH-31 Open Formula Mouse/Rat diet 7013 from Envigo, Madison, Wisconsin (hereafter referred to as the “chow” diet). 72 hrs after acclimatization, feeding with a ω6-rich diet (either diet 28 or 21, as indicated) or with the ω3-rich (diet 29) commenced. These isocalorific diets were modifications of the AIN-93G mouse diet, and were formulated to contain 3.8 Kcal/g with 19% of calories from protein (casein), 64% from carbohydrates, and 17% from fat (Envigo Teklad, Madison WI). The components of the diets are presented in Supplementary Table S1. Mice consumed equal amounts of calories per day on each diet. The food was changed twice per week. The diets were stored in vacuum-sealed pouches at −80 °C until used. Water was provided ad libitum. All the animals were kept in a pathogen-free environment in the animal facility.

### Mouse tumor models

Mice were divided randomly into groups. They were fed ad libitum with the experimental diets for 3 weeks prior to cell injection. Where indicated, TPPU was included in the water at 6.7 μg/L and the water changed twice weekly for the duration of the experiment. 8-week old C57BL/6 nude mice and C57BL/6 wild-type mice were injected i.p. with TCDD (at doses described below) in 10 μL1,4-dioxane, or with vehicle three days prior to cell injection. 1,4 dioxane was used as solvent for TCDD, rather than the commonly used corn oil, as the latter contains high levels of ω6 PUFA and its use would have seriously distorted the ω6/ω3 intake in the mice. C57BL/6 nude or wild-type mice were subcutaneously injected in the right flank with of single-cell suspensions of 10^6^ Hepa1-GFP cells or 2 × 10^5^ LLC cells respectively. Hepa1-GFP-treated C57BL/6 nude mice and LLC-treated C57BL/6 wild-type mice were injected i.p. weekly thereafter with 10 μL TCDD in 1,4-dioxane or vehicle until the end of the experiment, Beginning approximately two weeks after cell injection, tumor volume was assessed with an electronic vernier caliper every other day until the termination of the experiment or until resection of the tumor. If a mouse in any group died or had to be resected, the tumor volume data for all mice in the experiment were excluded from the tumor growth curves from that time forward so as to prevent biasing of the growth curves. Tumor volumes were determined using the formula: volume = (the shortest diameter)^2^ ×(the largest diameter)/2 × (4.19). For metastasis determinations, the complete primary tumors were surgically removed under isoflurane anesthesia when the tumor volumes were 2000–3500 mm^3^. (The range of volumes for any particular experiment are stated in the text). The wounds were closed with surgical staples, and the tumors were weighed. Gabapentin (100 mg/kg orally) plus caprofen (5 mg/kg subcutaneously) were used as analgesics immediately after surgery, daily thereafter for three days, and then as needed. Mice were euthanized approximately three to five weeks after surgery, their lungs weighed and their tissues examined for metastases (unless they were euthanized for health reasons or because of recurrence of the primary tumor, in which case they did not contribute to the metastasis data). Tumor tissue was fixed in formalin buffered saline (Fisherbrand, Pittsburg, PA), and embedded in paraffin for immunohistochemical analysis. The experiments were approved by the UCLA Institutional Animal Care and Use Committee (IACUC), and animals were cared for in accordance with institutional guidelines, and conformed with the guidelines of *Scientific Reports* in the following link www.nature.com/srep/policies/index.html#experimental-subjects

#### Lipid analysis

Fatty acids were analyzed after conversion to methyl esters (FAME) by gas chromatograph (GC)^47^. Extraction of oxylipins and their measurement by liquid chromatography with tandem mass spectrometry were performed as described previously^20^.

### Quantitative real time PCR and digital droplet PCR

Quantitative real time PCR, RNA was performed as previously described^46^. Means of three technical replicates were calculated and the data are presented for one of two or more representative independent experiments. Primers for qPCR were designed using Primer Express 3.0 software (Applied Biosystems, CA), and were synthesized by Thermo Fisher Scientific, Inc. (Waltham, MA). The primers for real time PCR are presented in Supplementary Table S5. Digital droplet PCR (ddPCR) was used to determine the amount of EPHX2 mRNA in the cells. 36B4 was used as a positive control. This procedure gives absolute numbers of molecules allowing one to express RNA concentrations as molecules of RNA per cell. cDNAs were generated using Clontech RNA to cDNA EcoDry Premix (Cat# 639549 Clontech, Mountain View, CA), and amplification droplets were generated on a Bio-Rad QX200 Droplet Generator using Bio-Rad ddPCR Supermix for Probes (no dUTP) (Cat# 1863024 Bio-Rad, Hercules, CA) and TaqMan hydrolysis probes labeled with fluorescein (FAM) or hexachlorofluorescein (HEX) reporter fluorophores purchased from Integrated DNA Technologies (IDT, Coralville, Iowa). PCR was performed on a deep well Bio Rad thermocycler using standard amplification procedures, and droplets were read on a BioRad QX200 Droplet Reader. Each cell extract was assayed at three different concentrations and the data calculated as the mean of these determinations. Dilutions that generated readings outside of the dynamic range were excluded. The primers and probes for ddPCR are presented in Supplementary Table S5.

### Immunohistochemical (IHC) analysis

Tissue blocks were cut and stained using the Vectastain Elite ABC HRP Kit (Vector; Burlingame, CA). Sections were deparaffinized in xylene, and dehydrated with alcohol. 3% hydrogen peroxide was used to block endogenous peroxidase activity. Antigen retrieval was performed and slides were treated with primary antibodies against CYP1A1 (1:1000 dilution) from Invitrogen (Carlsbad, CA), EPHX2 (1:750 dilution) from Novus Biologicals (Littleton, CO), VEGF (1:1500) from Abcam (Cambridge, MA), CD31 (1:1000) from Abcam, MCM2 (1:1500) from Abcam. Finally, sections were incubated with the Vectastain Elite ABC HRP Kit Secondary Antibody and DAB detection system from Vector Laboratories, Burlingame, CA, counterstained with hematoxylin, dehydrated, cleared, and mounted. Optimization of the IHC protocol involved three antigen-retrieval conditions and a serial dilution of the antibody to establish the optimal staining concentration. The IgG control was performed by using normal IgG rabbit Millipore (Temecula, CA). Apotosis was evaluated in tumor tissue samples using the *In Situ* Cell Death Detection Kit, (HRP) (Roche Applied Science, Mannheim, Germany).

The IHC-stained sections were digitized using an Aperio ScanScope CS (Leica, Nussloch, Germany) which produces 40× digital images with high resolution (0.45 um/pixel) as previously described^48^. Images were viewed and quantitated using an ImageScope (Aperio)^49^. Data are presented as total density/um^2^ analyzed in a total area of 10,000 µm^2^.

### VEGF ELISA

The Quantikine Mouse VEGF ELISA kit (R&D Systems, Minneapolis, MN) was used. Measurements were performed on three samples for each experimental condition. VEGF concentration was determined spectrophotometrically (Microplate Reader 680 XR; Bio-Rad, Hercules, CA) at 450 nm. In each experiment, all samples and standards were measured twice. Data were collected as optical density for each mouse serum (5 mice per group) and averaged.

### Statistical analysis

The continuous data from mouse experiments are presented as the mean +/− standard error of the mean, and analyzed using a 2-tailed Mann-Whitney U test for comparison of two independent groups. Fisher’s exact test was used for analysis of contingency tables. GraphPad Prism software (San Diego, CA) was used. Levels of significance are indicated as follows: *p < 0.05, **p<0.01, ***p<0.001.

## Supplementary information

Supplementary information.

Supplementary information.

Supplementary information2.

## Data Availability

All data generated or analysed during this study are included in the published article (and its supplementary information files) or are available from the corresponding author on reasonable request.

## References

[1] Kollcuri SK, Jin UH, Safe S (2017). Role of the aryl hydrocarbon receptor in carcinogenesis and potential as an anti-cancer drug target. Arch. Toxicol..

[2] Roager HM, Licht TR (2018). Microbial tryptophan catabolites in health and disease. Nat. Commun..

[3] Gutiérrez-Vázquez C, Quintana FJ (2018). Regulation of the immune response by the aryl hydrocarbonreceptor. Immunity..

[4] Murray IA, Patterson AD, Perdew GH (2014). Aryl hydrocarbon receptor ligands in cancer: friend and foe. Nat. Rev. Center.

[5] Forgacs AL (2012). Comparative metabolomic and genomic analyses of TCDD-elicited metabolic disruption in mouse and rat liver. Toxicol. Sci.

[6] Prokopec SD (2017). Compendium of TCDD-mediated transcriptomic response datasets in mammalian model systems. BMC Genomics..

[7] Díaz- Díaz CJ (2016). The aryl hydrocarbon receptor mediates the chemopreventive effect of indole-3-carbinol in an inflmmation-associated colorectal tumorigenesis model. Ann. Surg..

[8] Kawajiri K (2009). Aryl hydrocarbon receptor suppresses intestinal carcinogenesis in ApcMin/+ mice with natural ligands. PNAS..

[9] Huerta-Yepez S, Tirado-Rodriguez AB, Hankinson O (2016). Role of diets rich in omega-3 and omega-6 in the development of cancer. Bol. Med. Hosp. Infant. Mex..

[10] Zhang G, Kodani S, Hammock BD (2014). Stabilized epoxygenated fatty acids regulate inflammation, pain, angiogenesis and cancer. Prog. Lipid Res..

[11] Pozzi A (2010). The anti-tumorigenic properties of peroxisomal proliferator-activated receptor alpha are arachidonic acid epoxygenase-mediated. J. Biol. Chem..

[12] Panigrahy D (2012). Epoxyeicosanoids stimulate multiorgan metastasis and tumor dormancy escape in mice. J. Clin. Invest..

[13] Zhang G (2013). Epoxy metabolites of docosahexaenoic acid (DHA) inhibit angiogenesis, tumor growth, and metastasis. PNAS..

[14] Jiang JG (2005). Cytochrome P450 2J2 promotes the neoplastic phenotype of carcinoma cells and is up-regulated in human tumors. Cancer Res..

[15] Jiang JG (2007). Cytochrome p450 epoxygenase promotes human cancer metastasis. Cancer Res..

[16] Choudhary D, Jansson I, Stoilov I, Sarfarazi M, Schenkman JB (2004). Metabolism of retinoids and arachidonic acid by human and mouse cytochrome P450 1b1. Drug Metab. Dispos..

[17] Fer MY (2008). Metabolism of eicosapentaenoic and docosahexaenoic acids by recombinant human cytochromes P450. Arch. Biochem. Biophys.

[18] Schwartz D (2004). Arachidonic and eicosapentaenoic acid metabolism by human CYP1A1: highly stereoselective formation of 17(R),18(S)-epoxyeicosatetraenoic acid. Biochem. Pharmacol..

[19] Bui P, Solaimani P, Wu X, Hankinson O (2012). 2,3,7,8-Tetrachlorodibenzo-p-dioxin treatment alters eicosanoid levels in several organs of the mouse in an aryl hydrocarbon receptor-dependent fashion. Toxicol. Appl. Pharmacol..

[20] Yang J, Solaimani P, Dong H, Hammock BD, Hankinson O (2013). Treatment of mice with 2,3,7,8-Tetrachlorodibenzo-p-dioxin markedly increases the levels of a number of cytochrome P450 metabolites of omega-3 polyunsaturated fatty acids in the liver and lung. J. Toxicol. Sci..

[21] Simopoulos AP (2002). The importance of the ratio of omega-6/omega-3 essential fatty acids. Biomed. Pharmacother..

[22] Abel J, Li W, Döhr O, Vogel C, Donat S (1996). Dose-response relationship of cytochrome P4501b1 mRNA induction by 2,3,7,8-tetrachlorodibenzo-p-dioxin in livers of C57BL/6J and DBA/2J mice. Arch. Toxicol..

[23] Boverhof DR (2005). Temporal and dose-dependent hepatic gene expression patterns in mice provide new insights into TCDD-Mediated hepatotoxicity. Toxicol. Sci.

[24] Yang YM (2018). Estrogen-dependent epigenetic regulation of soluble epoxide hydrolase via DNA methylation. PNAS..

[25] Angrish MM, Dominici CY, Zacharewski TR (2013). TCDD-elicited effects on liver, serum, and adipose lipid composition in C57BL/6 mice. Toxicol. Sci.

[26] Zhang L (2015). Metabolomics reveals that aryl hydrocarbon receptor activation by environmental chemicals induces systemic metabolic dysfunction in mice. Environ. Sci. Technol..

[27] Mastrangelo G, Fadda E, Marzia V (1996). Polycyclic aromatic hydrocarbons and cancer in man. Environ. Health Perspect..

[28] Kim KH, Jahan SA, Kabir E, Brown RJ (2013). A review of airborne polycyclic aromatic hydrocarbons (PAHs) and their human health effects. Environ. Intl.

[29] Fontana RJ (1999). Effects of a chargrilled meat diet on expression of CYP3A, CYP1A, and P-glycoprotein levels in healthy volunteers. Gastroenterology..

[30] Knize MG, Salmon CP, Pais P, Felton JS (1999). Food heating and the formation of heterocyclic aromatic amine and polycyclic aromatic hydrocarbon mutagens/carcinogens. Adv. Exp. Med. Biol.

[31] Seok SH (2018). Trace derivatives of kynurenine potently activate the aryl hydrocarbon receptor (AHR). J. Biol. Chem..

[32] Opitz CA (2011). An endogenous tumour-promoting ligand of the human aryl hydrocarbon receptor. Nature..

[33] Androutsopoulos, V.P., Tsatsakis, A.M., & Spandidos, D.A. Cytochrome P450 CYP1A1: wider roles in cancer progression and prevention. *BMC Cancer*. **9**(187) (2009). 10.1186/1471-2407-9-187 PMC2703651 19531241

[34] Safe S, Lee SO, Jin UH (2013). Role of the aryl hydrocarbon receptor in carcinogenesis and potential as a drug target. Toxicol. Sci.

[35] Go RE, Hwang KA, Choi KC (2014). Cytochrome P450 1 family and cancers. J. Steroid Biochem. Mol. Bio.

[36] Enayetallah AE, French RA, Grant DF (2006). Distribution of soluble epoxide hydrolase, cytochrome P450 2C8, 2C9 and 2J2 in human malignant neoplasms. J. Mol. Hist.

[37] Przybyla-Zawislak BD (2003). Polymorphisms in human soluble epoxide hydrolase. Mol. Pharmacol..

[38] Yang J (2017). Postprandial effect to decrease soluble epoxide hydrolase activity: roles of insulin and gut microbiota. J. Nutr. Biochem. 2017.

[39] Keserü B (2010). Hypoxia-induced pulmonary hypertension: comparison of soluble epoxide hydrolase deletion vs. inhibition. Cardiovasc Res.

[40] Baker EJ, Miles EA, Burdge GC, Yaoob P, Calder PC (2016). Metabolism and functional effects of plant-derived omega-3 fatty acids in humans. Prog. Lipid Res..

[41] Mollerup S, Ryberg D, Hewer A, Phillips DH, Haugen A (1999). Sex differences in lung CYP1A1 expression and DNA adduct levels among lung cancer patients. Cancer Res..

[42] Spivack SD, Hurteau GJ, Fasco MJ, Kaminsky LS (2003). Phase I and II carcinogen and metabolism gene expression in human lung tissue and tumors. Clin. Cancer Res..

[43] Khan WA (2017). Bioengineered plants can be a userful source of omega-3 fatty acids. Biomed Res. Int..

[44] Rose TE (2010). 1-Aryl-3-(1-acylpiperidin-4-yl)urea inhibitors of human and murine soluble epoxide hydrolase: structure – activity relationships, pharmacokinetics, and reduction of inflammatory pain. J. Med. Chem..

[45] Hankinson O (1979). Single-step selection of clones of a mouse hepatoma line deficient in aryl hydrocarbon hydroxylase. PNAS..

[46] Solaimani P, Damoiseaux R, Hankinson O (2013). Genome-wide RNAi high-throughput screen identifies proteins necessary for the AHR-dependent induction of CYP1A1 by 2,3,7,8-Tetrachlorodibenzo-p-dioxin. Toxicol. Sci.

[47] Muñiz-Hernández S (2016). Association between nuclear expression of retinoic acid receptor alpha and beta and clinicopathological features and prognosis of advanced non-small cell lung cancer. Int. J. Clin. Oncol..

[48] Laurinaviciene A (2014). Digital immunohistochemistry platform for the staining variation monitoring based on integration of image and statistical analyses with laboratory information system. Diagn. Pathol..

