# Supplementary material for: Aryl Hydrocarbon Receptor-Dependent inductions of omega-3 and omega-6 polyunsaturated fatty acid metabolism act inversely on tumor progression
Source: Sci Rep. 2020 May 12;10:7843. doi: 10.1038/s41598-020-64146-6 (PMC7217871; doi:10.1038/s41598-020-64146-6)
Supplement: Supplementary file 1 — Supplementary information. [file 41598_2020_64146_MOESM1_ESM.docx]

**Supplementary Material**

Aryl Hydrocarbon Receptor-Dependent inductions of omega-3 and omega-6 polyunsaturated fatty acid metabolism act inversely on tumor progression

Sara Huerta-Yepez, Ana Tirado-Rodriguez, Mayra R. Montecillo-Aguado, Jun Yang, Bruce D. Hammock and Oliver Hankinson

**Supplementary Table S1.** Compositions of experimental diets

|  | **TD.140429** | **TD.140428** | **TD.150321** |
| --- | --- | --- | --- |
| Component | g/Kg | g/Kg | g/Kg |
| Casein | 200.0 | 200.0 | 200.0 |
| L-Cystine | 3.0 | 3.0 | 3.0 |
| Corn Starch | 397.486 | 397.486 | 397.486 |
| Maltodextrin | 132.0 | 132.0 | 132.0 |
| Sucrose | 100.0 | 100.0 | 100.0 |
| Coconut Oil | 49.175 | 49.175 | 49.175 |
| Fish Oil | 14.3 | -- | 1.22 |
| Corn Oil | 6.525 | 20.825 | 19.605 |
| Cellulose | 50.0 | 50.0 | 50.0 |
| Mineral Mix, AIN-93G-MX (94046) | 35.0 | 35.0 | 35.0 |
| Vitamin Mix, AIN-93-VX (94047) | 10.0 | 10.0 | 10.0 |
| Choline Bitartrate | 2.5 | 2.5 | 2.5 |
| TBHQ, antioxidant | 0.014 | 0.014 | 0.014 |

**Supplementary Table S2**. Levels of individual PUFA in organs of mice 24 days after initiation of feeding of the diets. (pmol/gm tissue)


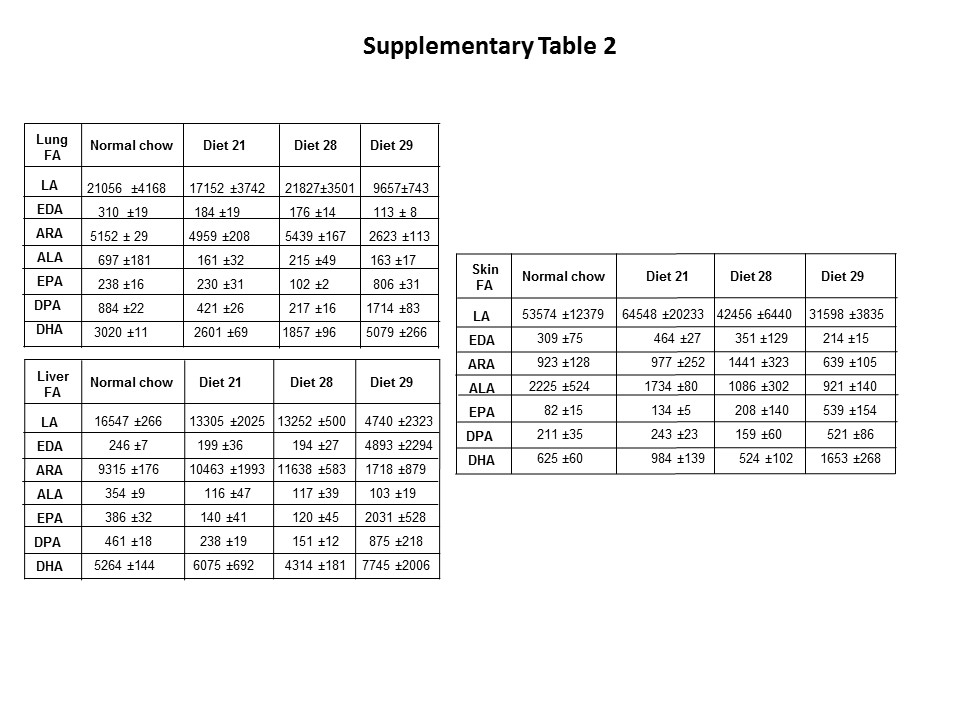


**Supplementary Table S3**. Levels of oxylipins in Hepa1-GFP-derived tumors, in lungs and in livers.

Please refer to the separate excel file. Data are in pmol/gm tissue.

**Supplementary Table S4**. The volumes and weights of tumors at resection. (A: Hepa1-GFP tumors, B to E: LLC tumors).


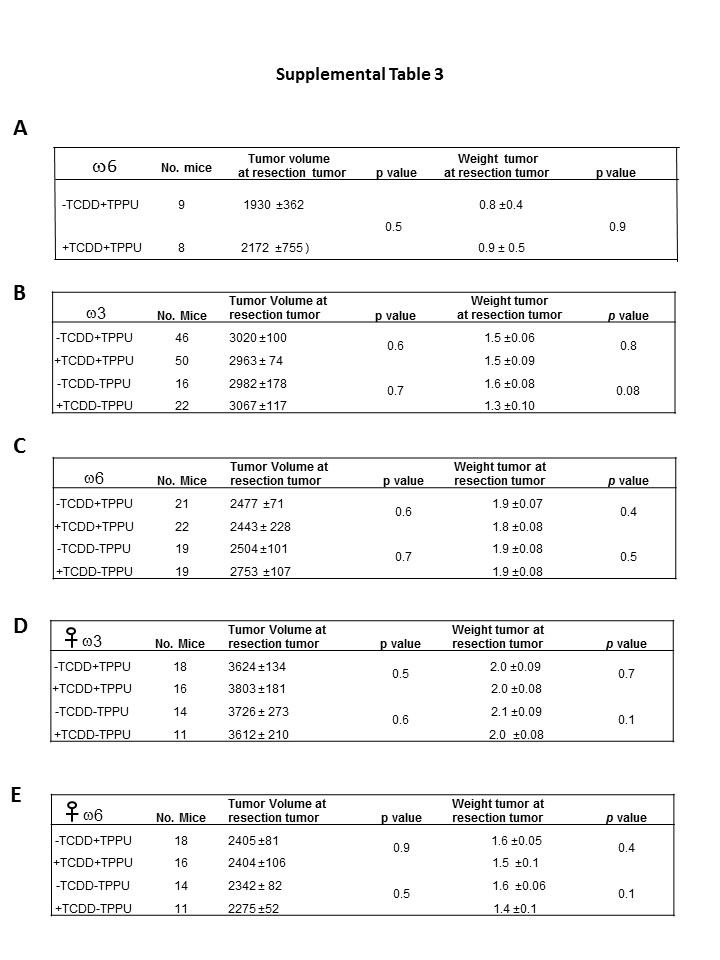


**Supplementary Table S5. Sequences of RT-PCR primers.**

| CYP1A1 | Forward (F)-AGAGCACTACAGGACATTTGAG  Reverse (R)-CCAAAGAGGTCCAAAACAATCG |
| --- | --- |
| CYP1A2 | F-TGGAGCTGGCTTTGACACAGT  R-GCCATGTCACAAGTAGCAAAATG |
| CYP1B1 | F-ACCAGAAGTCCTCCTACCAAGAGA  R-GCCTCATCCAGGGCTATAAAGG |
| EPHX2 | F-GGTTACCATCCTGGTCCACA  R-TGTGTCCCTGTGACCTTCTC |
| AHR primers (proprietary sequences) were purchased from Qiagen, Valencia, CA (catalog # PPM03973F-200) |  |
| Tubulin (TUBD1) mRNA was determined by Taqman Assays using TUBD1 probe from Applied Biosystems (Mm00444851_m1) |  |
| 36B4 | F-GGACCCGAGAAGACCTCCTT  R-GCACATCACTCAGAATTTCAA |
| EPHX2 (Digital droplet PCR primers) | F-GCCAGTGATGAGACAGGTTT  R-GCTGAGGTTGGGATCTTCTG |
| 36B4 (Digital droplet PCR primers) | F-ACCGCCTGGTTCTCCTATAA  R-AAGACGATGTCACTCCAACG |

**
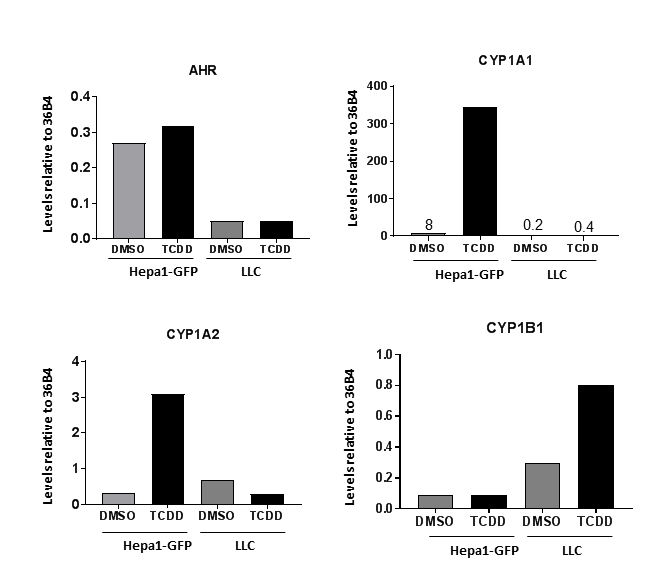
Supplementary Figure S1**. AHR and CYP1 mRNA expression in Hepa1-GFP and LLC cells treated for 3 days with 1 nM TCDD or 0.1% DMSO (vehicle). The values are represented relative to the constitutively expressed ribosomal protein 36B4, are the means of duplicate determinations in each case, and representative of at least three independent determinations.


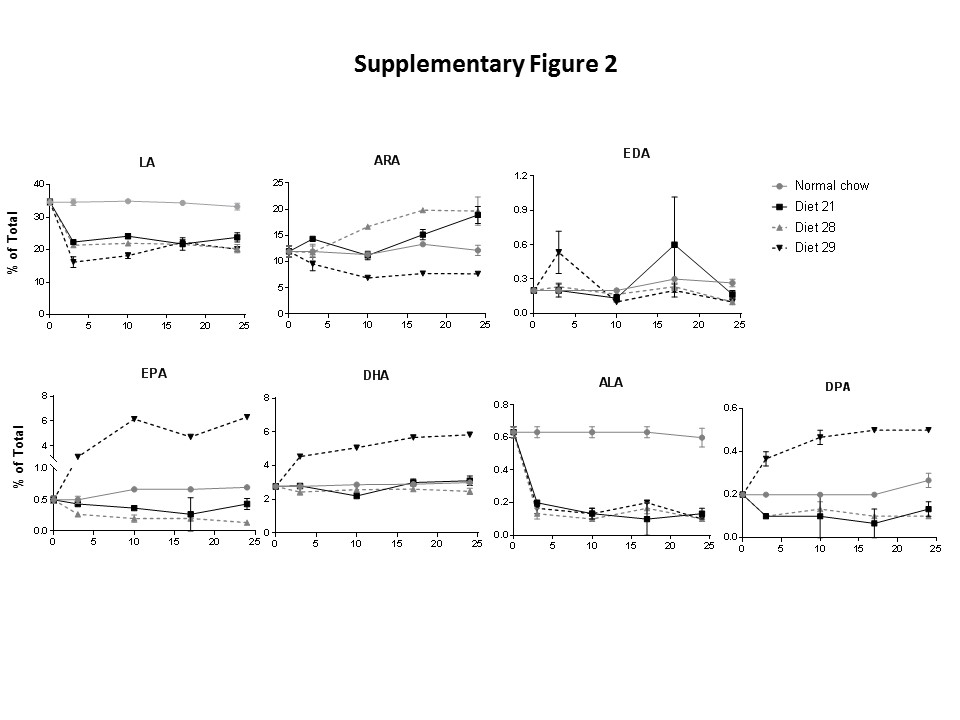


**Supplementary Figure S2**. Levels of individual PUFA in plasma of mice after initiation of feeding of the diets. EDA is Eicosadienoic acid and DPA is Docosapentenoic acid.


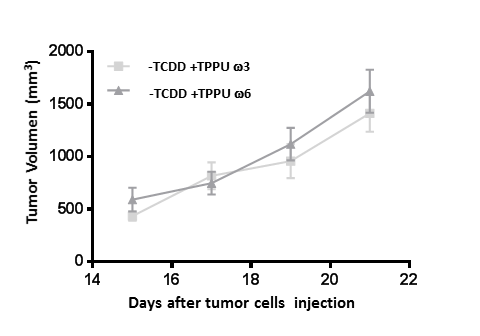


**Supplementary Figure S3**. Tumor growth rates of LLC-derived tumors in mice fed diet 29 ω3-rich and ω6-rich diet 21 in the absence of TCDD. There were no significant differences at any time point. A representative experiment of four independent experiments is shown.

**
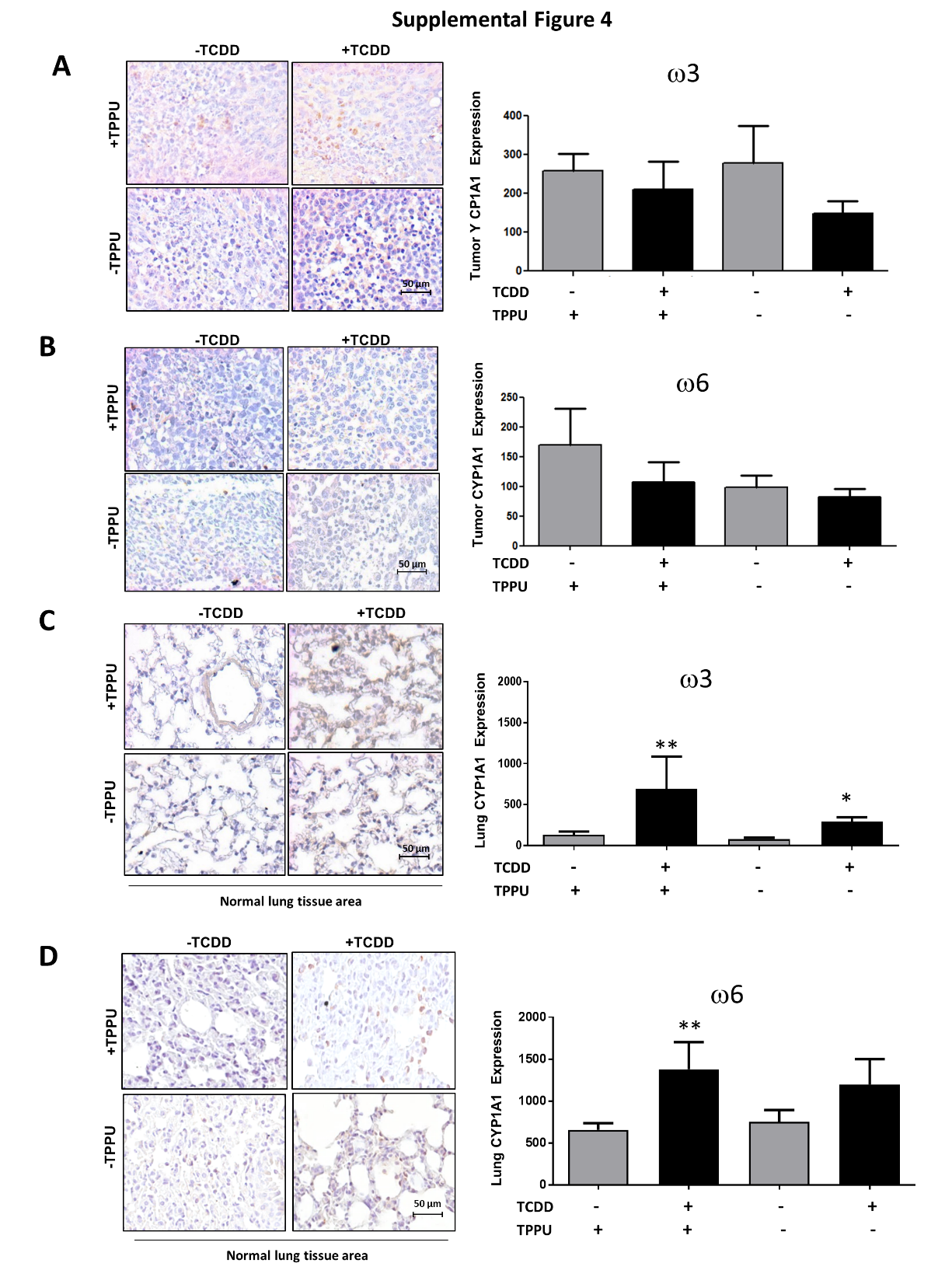
a**

**b**

**c**

**d**

**Supplementary Figure S4.** CYP1A1 protein levels in LLC-derived tumors from male mice fed the ω3-rich (A) or ω6-rich (B) diets, and in non-metastatic segments of the lungs of male mice fed with the ω3-rich (C) and ω6-rich (D) diets.

**
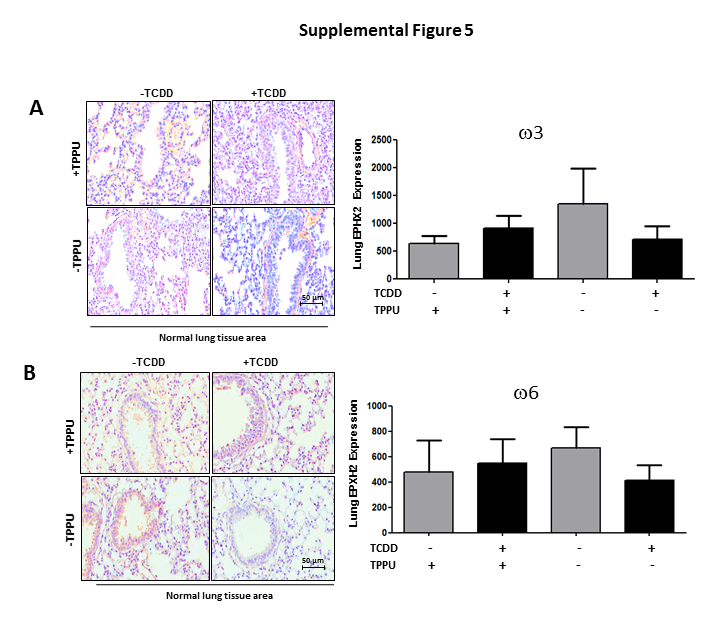
a**

**b**

**Supplementary Figure S5**. EPHX2 levels in lung.
