# Supplementary material for: Aryl Hydrocarbon Receptor-Dependent inductions of omega-3 and omega-6 polyunsaturated fatty acid metabolism act inversely on tumor progression
Source: Sci Rep. 2020 May 12;10:7843. doi: 10.1038/s41598-020-64146-6 (PMC7217871; doi:10.1038/s41598-020-64146-6)
Supplement: Supplementary file 2 — Supplementary information. [file 41598_2020_64146_MOESM2_ESM.pdf]

Supplementary Material

Aryl Hydrocarbon Receptor-Dependent inductions of omega-3 and omega-6 polyunsaturated fatty acid metabolism act inversely on tumor progression

Sara Huerta-Yepez[1], Ana Tirado-Rodriguez[1], Mayra R. Montecillo-Aguado[2], Jun Yang[3], Bruce D. Hammock[3] and *Oliver Hankinson[1,4]

[1]Department of Pathology & Laboratory Medicine, University of California, Los Angeles, CA 90095
[2]Research Unit of Oncology Diseases. Hospital Infantil de Mexico, Federico Gomez. Mexico City, Mexico.
[3]Department of Entomology and Comprehensive Cancer Center, University of California, Davis, CA 95616
[4]Molecular Toxicology Interdepartmental Program and Department of Environmental Health Sciences, University of California, Los Angeles, CA 90095

Address correspondence to:
*Oliver Hankinson, Department of Pathology & Laboratory Medicine, UCLA Medical Center, Center for the Health Sciences Box 951732, Los Angeles, CA 90095-1732, ohank@mednet.ucla.edu

Supplementary Table S1. Compositions of experimental diets

	TD.140429	TD.140428	TD.150321
Component	g/Kg	g/Kg	g/Kg
Casein	200.0	200.0	200.0
L-Cystine	3.0	3.0	3.0
Corn Starch	397.486	397.486	397.486
Maltodextrin	132.0	132.0	132.0
Sucrose	100.0	100.0	100.0
Coconut Oil	49.175	49.175	49.175
Fish Oil	14.3	--	1.22
Corn Oil	6.525	20.825	19.605
Cellulose	50.0	50.0	50.0
Mineral Mix, AIN-93G-MX (94046)	35.0	35.0	35.0
Vitamin Mix, AIN-93-VX (94047)	10.0	10.0	10.0
Choline Bitartrate	2.5	2.5	2.5
TBHQ, antioxidant	0.014	0.014	0.014

Supplementary Table S2. Levels of individual PUFA in organs of mice 24 days after initiation of feeding of the diets. (mol/gm tissue)

Lung FA	Normal chow	Diet 21	Diet 28	Diet 29
LA	21056 ±4168	17152 ±3742	21827±3501	9657±743
EDA	310 ±19	184 ±19	176 ±14	113 ± 8
ARA	5152 ± 29	4959 ±208	5439 ±167	2623 ±113
ALA	697 ±181	161 ±32	215 ±49	163 ±17
EPA	238 ±16	230 ±31	102 ±2	806 ±31
DPA	884 ±22	421 ±26	217 ±16	1714 ±83
DHA	3020 ±11	2601 ±69	1857 ±96	5079 ±266

Liver FA	Normal chow	Diet 21	Diet 28	Diet 29
LA	16547 ±266	13305 ±2025	13252 ±500	4740 ±2323
EDA	246 ±7	199 ±36	194 ±27	4893 ±2294
ARA	9315 ±176	10463 ±1993	11638 ±583	1718 ±879
ALA	354 ±9	116 ±47	117 ±39	103 ±19
EPA	386 ±32	140 ±41	120 ±45	2031 ±528
DPA	461 ±18	238 ±19	151 ±12	875 ±218
DHA	5264 ±144	6075 ±692	4314 ±181	7745 ±2006

Skin FA	Normal chow	Diet 21	Diet 28	Diet 29
LA	53574 ±12379	64548 ±20233	42456 ±6440	31598 ±3835
EDA	309 ±75	464 ±27	351 ±129	214 ±15
ARA	923 ±128	977 ±252	1441 ±323	639 ±105
ALA	2225 ±524	1734 ±80	1086 ±302	921 ±140
EPA	82 ±15	134 ±5	208 ±140	539 ±154
DPA	211 ±35	243 ±23	159 ±60	521 ±86
DHA	625 ±60	984 ±139	524 ±102	1653 ±268

Supplementary Table S3. Levels of oxylipins in Hepa1-GFP-derived tumors, in lungs and in livers.

Please refer to the separate excel file

Supplementary Table S4. The volumes and weights of tumors at resection. (A: Hepa1-GFP tumors, B to E: LLC tumors).

A

ω6	No. mice	Tumor volume at resection tumor	p value	Weight tumor at resection tumor	p value
-TCDD+TPPU	9	1930 ±362	0.5	0.8 ±0.4	0.9
+TCDD+TPPU	8	2172 ±755)		0.9 ± 0.5	

B

ω3	No. Mice	Tumor Volume at resection tumor	p value	Weight tumor at resection tumor	*p* value
-TCDD+TPPU	46	3020 ±100	0.6	1.5 ±0.06	0.8
+TCDD+TPPU	50	2963 ± 74		1.5 ±0.09	
-TCDD-TPPU	16	2982 ±178	0.7	1.6 ±0.08	0.08
+TCDD-TPPU	22	3067 ±117		1.3 ±0.10	

C

ω6	No. Mice	Tumor Volume at resection tumor	p value	Weight tumor at resection tumor	*p* value
-TCDD+TPPU	21	2477 ±71	0.6	1.9 ±0.07	0.4
+TCDD+TPPU	22	2443 ± 228		1.8 ±0.08	
-TCDD-TPPU	19	2504 ±101	0.7	1.9 ±0.08	0.5
+TCDD-TPPU	19	2753 ±107		1.9 ±0.08	

D

♀ ω3	No. Mice	Tumor Volume at resection tumor	p value	Weight tumor at resection tumor	*p* value
-TCDD+TPPU	18	3624 ±134	0.5	2.0 ±0.09	0.7
+TCDD+TPPU	16	3803 ±181		2.0 ±0.08	
-TCDD-TPPU	14	3726 ± 273	0.6	2.1 ±0.09	0.1
+TCDD-TPPU	11	3612 ± 210		2.0 ±0.08	

E

♀ ω6	No. Mice	Tumor Volume at resection tumor	p value	Weight tumor at resection tumor	*p* value
-TCDD+TPPU	18	2405 ±81	0.9	1.6 ±0.05	0.4
+TCDD+TPPU	16	2404 ±106		1.5 ±0.1	
-TCDD-TPPU	14	2342 ± 82	0.5	1.6 ±0.06	0.1
+TCDD-TPPU	11	2275 ±52		1.4 ±0.1	

Supplementary Table S5. Sequences of RT-PCR primers.

CYP1A1	Forward (F)-AGAGCACTACAGGACATTTGAG<br>Reverse (R)-CCAAAGAGGTCCAAAACAATCG
CYP1A2	F-TGGAGCTGGCTTTGACACAGT<br>R-GCCATGTCACAAGTAGCAAAATG
CYP1B1	F-ACCAGAAGTCCTCCTACCAAGAGA<br>R-GCCTCATCCAGGGCTATAAAGG
EPHX2	F-GGTTACCATCCTGGTCCACA<br>R-TGTGTCCCTGTGACCTTCTC
AHR primers (proprietary sequences) were purchased from Qiagen, Valencia, CA (catalog # PPM03973F-200)	
Tubulin (TUBD1) mRNA was determined by Taqman Assays using TUBD1 probe from Applied Biosystems (Mm00444851_m1)	
36B4	F-GGACCCGAGAAGACCTCCTT<br>R-GCACATCACTCAGAATTTCAA
EPHX2 (Digital droplet PCR primers)	F-GCCAGTGATGAGACAGGTTT<br>R-GCTGAGGTTGGGATCTTCTG
36B4 (Digital droplet PCR primers)	F-ACCGCCTGGTTCTCCTATAA<br>R-AAGACGATGTCACTCCAACG

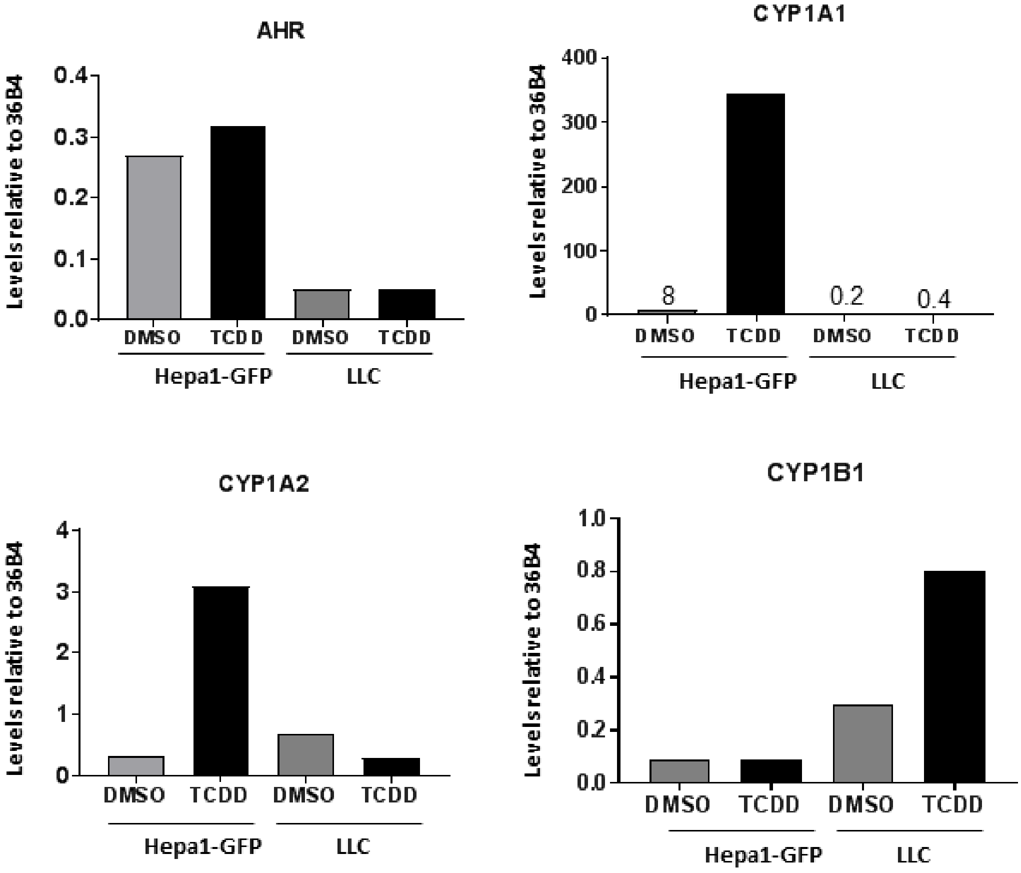

Supplementary Figure S1. AHR and CYP1 mRNA expression in Hepa1-GFP and LLC cells treated for 3 days with 1 nM TCDD or 0.1% DMSO (vehicle). The values are represented relative to the constitutively expressed ribosomal protein 36B4, are the means of duplicate determinations in each case, and representative of at least three independent determinations.

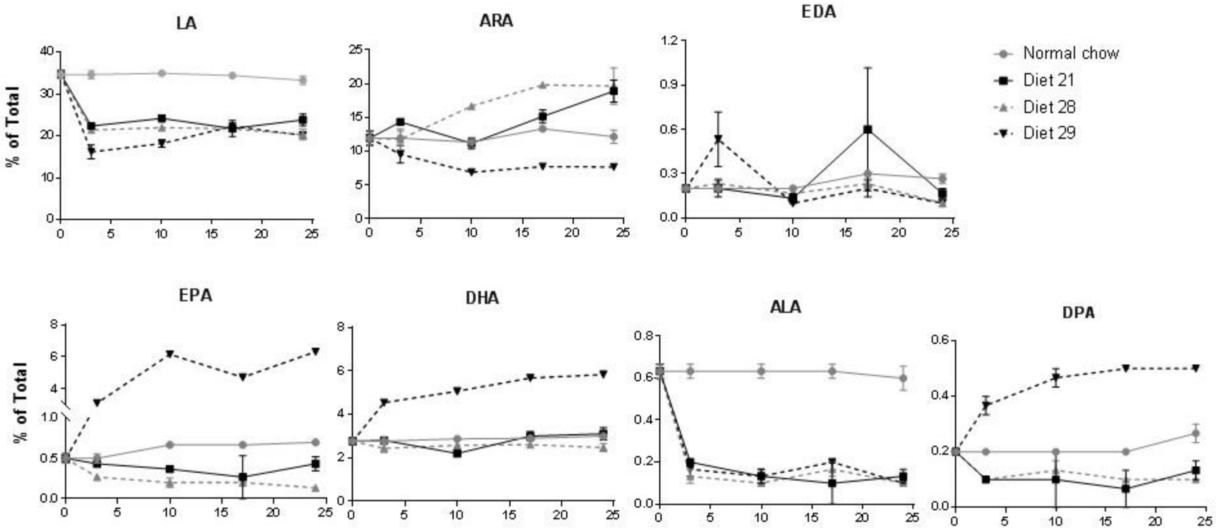

Supplementary Figure S2. Levels of individual PUFA in plasma of mice after initiation of feeding
of the diets. EDA is Eicosadienoic acid and DPA is Docosapentenoic acid.

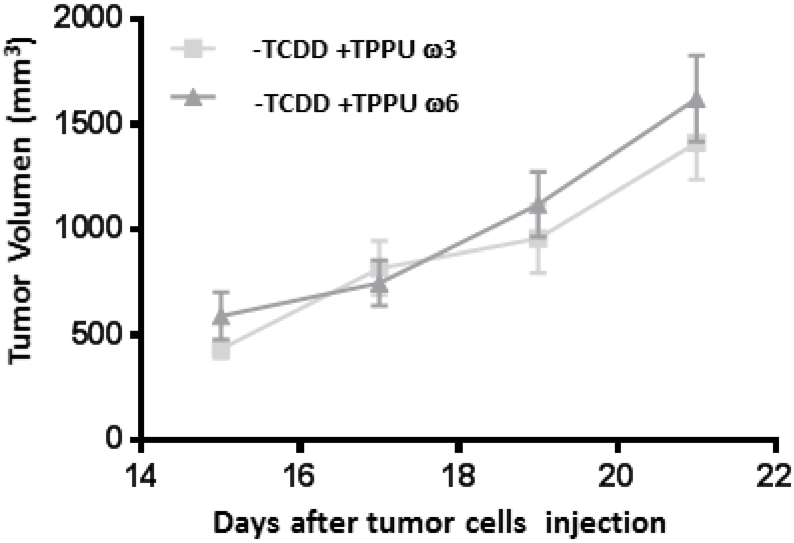

Supplementary Figure S3. Tumor growth rates of LLC-derived tumors in mice fed diet 29 ω3-rich and ω6-rich diet 21 in the absence of TCDD. There were no significant differences at any time point. A representative experiment of four independent experiments is shown.

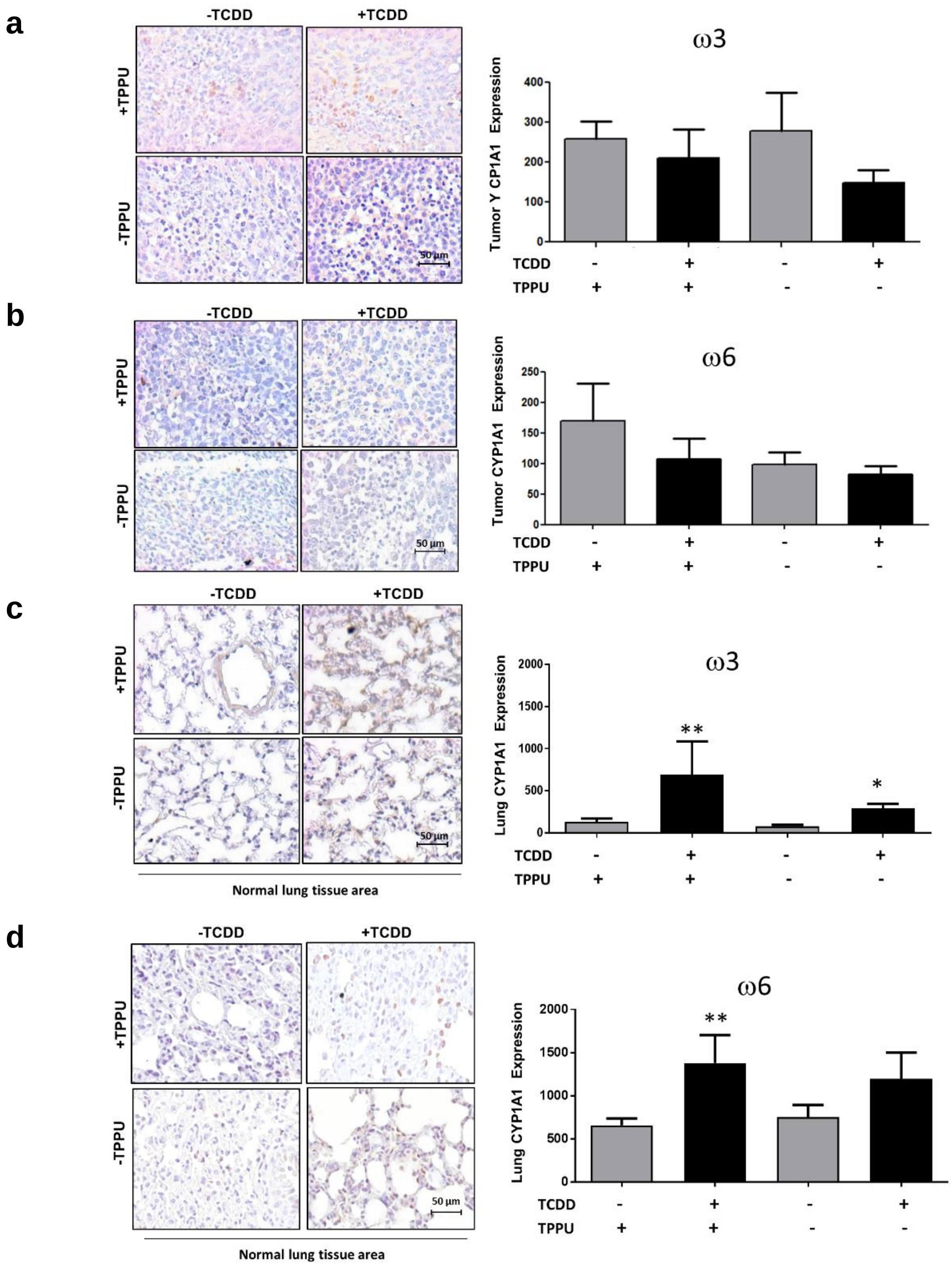

Supplementary Figure S4. CYP1A1 protein levels in LLC-derived tumors from male mice fed the ω3-rich (A) or ω6-rich (B) diets, and in non-metastatic segments of the lungs of male mice fed with the ω3-rich (C) and ω6-rich (D) diets.

a

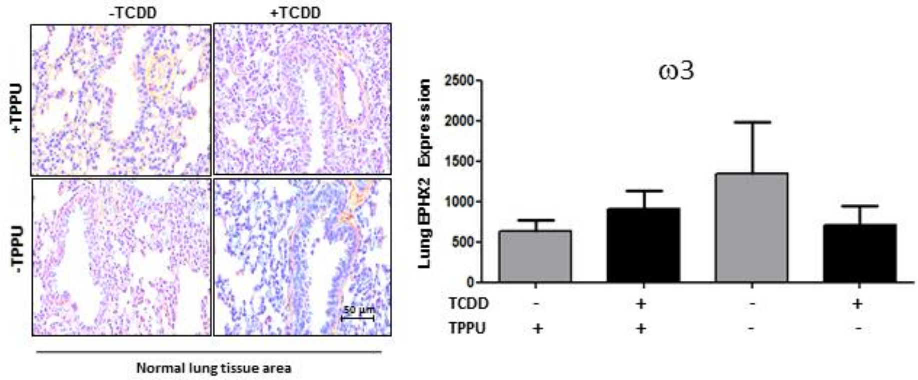

b

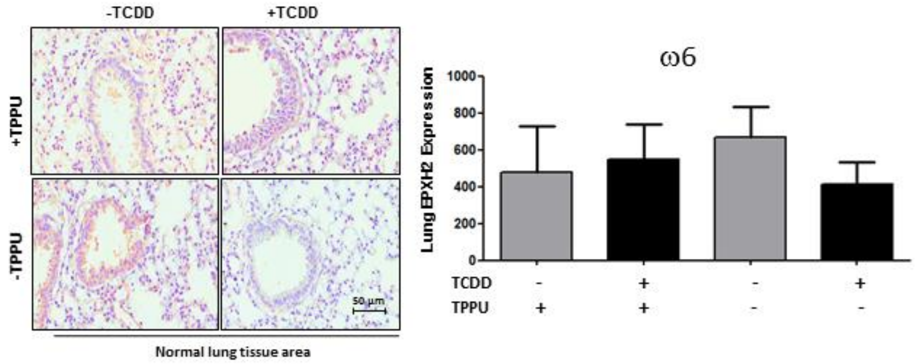

Supplementary Figure S5. EPHX2 levels in lung.
